# ApcE plays an important role in light-induced excitation energy dissipation in the *Synechocystis* PCC6803 phycobilisomes

**DOI:** 10.1007/s11120-024-01078-6

**Published:** 2024-02-26

**Authors:** Gonfa Tesfaye Assefa, Joshua L. Botha, Bertus van Heerden, Farooq Kyeyune, Tjaart P. J. Krüger, Michal Gwizdala

**Affiliations:** 1 Department of Physics, University of Pretoria, Lynnwood Road, Pretoria, 0002 South Africa; 2 Forestry and Agricultural Biotechnology Institute (FABI), University of Pretoria, Lynnwood Road, Pretoria, 0002 South Africa; 3 National Institute for Theoretical and Computational Sciences (NITheCS), Stellenbosch, South Africa; 4 Present Address: Department of Physics, Faculty of Science, Kyambogo University, P.O. Box 1, Kyambogo, Kampala, Uganda; 5 Present Address: ICFO - Institut de Ciencies Fotoniques, The Barcelona Institute of Science and Technology, Castelldefels, 08860 Spain

**Keywords:** Single-Molecule Spectroscopy, Phycobilisomes, Excitation Energy Transfer, Thermal Energy Dissipation, Photoregulation

## Abstract

Phycobilisomes (PBs) play an important role in cyanobacterial photosynthesis. They capture light and transfer excitation energy to the photosynthetic reaction centres. PBs are also central to some photoprotective and photoregulatory mechanisms that help sustain photosynthesis under non-optimal conditions. Amongst the mechanisms involved in excitation energy dissipation that are activated in response to excessive illumination is a recently discovered light-induced mechanism that is intrinsic to PBs and has been the least studied. Here, we used single-molecule spectroscopy and developed robust data analysis methods to explore the role of a terminal emitter subunit, ApcE, in this intrinsic, light-induced mechanism. We isolated the PBs from WT *Synechocystis* PCC 6803 as well as from the ApcE-C190S mutant of this strain and compared the dynamics of their fluorescence emission. PBs isolated from the mutant (i.e., ApcE-C190S-PBs), despite not binding some of the red-shifted pigments in the complex, showed similar global emission dynamics to WT-PBs. However, a detailed analysis of dynamics in the core revealed that the ApcE-C190S-PBs are less likely than WT-PBs to enter quenched states under illumination but still fully capable of doing so. This result points to an important but not exclusive role of the ApcE pigments in the light-induced intrinsic excitation energy dissipation mechanism in PBs.

**Supplementary Information:**

The online version contains supplementary material available at 10.1007/s11120-024-01078-6.

## Introduction

Cyanobacteria and some algae use phycobilisomes (PBs) as their major photosynthetic light-harvesting complexes (LHCs), transferring excitation energy to the reaction centres (RCs). PBs are water-soluble, multi-subunit pigment-protein complexes adherent to the cytoplasmic (stromal) surface of thylakoid membranes (Glazer 1984). While their general architecture can differ depending on the specific organism or conditions, all types of PBs bind numerous linear tetrapyrrole pigments (Zhao et al. 2012). PBs are all composed of multiple pigment-protein subunits and (mostly) non-pigmented linker proteins (de Marsac and Cohen-bazire 1977) that work as a scaffold and tune the optical properties of the pigmented subunits (Adir 2005). Pigment-protein subunits of PBs are organised as heterodimers – often referred to as “monomers” – that, in turn, form trimers and hexamers.

In the hemi-discoidal structure of *Synechocystis* PCC6803 (hereafter, *Synechocystis*) PBs, the tri-cylindrical core is surrounded by, on average, six rods (Arteni et al. 2009). Each rod is formed by two to three phycocyanin (PC) hexamers and a number of colour-less linker proteins that define the order of hexamers and tune the properties of the pigments. Each PC hexamer is composed of 12 polypeptides covalently binding 18 phycocyanobilin pigments (Yu et al. 1981; Duerring et al. 1991). Typically, a PC hexamer from *Synechocystis* absorbs light at ~ 620 nm and emits at ~ 650 nm, although red-shifted emission from PC was also reported (Gwizdala et al. 2018b). Rods are attached to the core via the rod-core linker proteins (Yamanaka et al. 1980; Lundell et al. 1981).

The primary building block of the core is the allophycocyanin (APC) trimer composed of three ApcA and three ApcB subunits—six polypeptides covalently binding six phycocyanobilin pigments (Zilinskas 1982; Lundell and Glazer 1983a, b). Four APC trimers and two small core linker proteins (ApcC) form the upper cylinder of the core (distal from the thylakoid membrane). With absorption at ~ 650 nm and emission at ~ 660 nm, APC trimers receive excitation energy from the PC rods and transfer it to the bottom cylinders of the core, which, in addition to ApcAs, ApcBs and ApcCs, also contain specialised subunits—ApcD, ApcE and ApcF—collectively referred to as terminal emitters (TEs) (Lundell and Glazer 1983a, b). While ApcD and ApcE replace single ApcA subunits in two adjacent APC trimers in the bottom cylinders, ApcF replaces an ApcB subunit in the trimer containing ApcE. A structural model of *Synechocystis’* PBs (Liu et al. 2021), based on chemical cross-linking studies and cryo-electron microscopy structures of red algae PBs (Zhang et al. 2017; Ma et al. 2020), as well as the recent structures of cyanobacterial phycobilisomes (Zheng et al. 2021; Domínguez-Martín et al. 2022), revealed that in *Synechocystis*, ApcE and ApcF do not form a heterodimer (“monomer”), as suggested previously (Lundell and Glazer 1983b), but instead belong to two neighbouring heterodimers. Thus, according to this model, their pigments are separated by the shortest distance between any phycocyanobilins in *Synechocystis’* PBs (20,6 Å) (Liu et al. 2021).

The optical properties of the TEs seem to differ across cyanobacterial strains, as evidenced by different optical properties of PBs isolated from different mutants and deprived of one or more TEs (e.g., ApcD or ApcF) (Maxson et al. 1989; Gindt et al. 1992, 1994; Ashby and Mullineaux 1999; Jallet et al. 2012). Moreover, the specific functions and roles in energy transfer of different TEs were also recently shown to vary across the strains (Calzadilla et al. 2019). However, there seems to be a consensus that in all strains, ApcE is a red-shifted component of PB with emission at ~ 680 nm and is responsible for transferring excitation energy to the photosynthetic RCs.

**Fig. 1:**
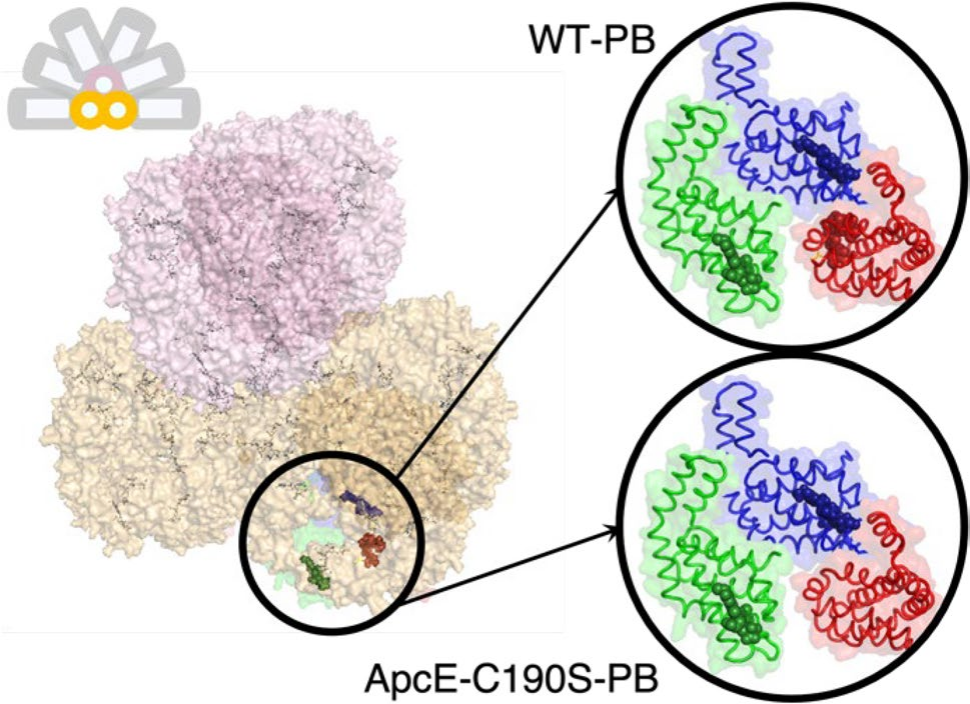
Structural model of the PB of *Synechocystis*, in which the rods surround the central core (inset in the upper left corner). The upper cylinder of the PB core is shown in pink, while the bottom cylinders are in orange. The TE subunits in one of the bottom APC cylinders in WT-PB and in ApcE-C190S-PB are shown in the upper and lower circles, respectively. The protein matrix is shown as a semi-transparent surface and the phycocyanobilin pigments are shown as sticks or, for the TE pigments, as spheres. TE subunits are ApcD (green), ApcF (blue), and ApcE (red). In WT-PBs, all TE subunits bind pigments. In ApcE-C190S-PB, cysteine 190 was replaced by a serine and ApcE does not covalently bind a pigment. Structures were obtained from the structural model of (Liu et al. 2021) and redrawn in PyMol 1.6

While the mutants missing genes encoding for the ApcD or ApcF still constitute functional PBs, with ApcA or ApcB replacing these missing subunits, respectively (Gindt et al. 1994; Ashby and Mullineaux 1999; Jallet et al. 2012), the lack of ApcE prevents the formation of a PB complex (Shen et al. 1993). Apart from its pigmented domain that resembles ApcA, ApcE also contains 1) a few linker domains that serve as a scaffold for other APC trimers in the core, and 2) a loop responsible for anchoring PBs to the thylakoid membranes (Capuano et al. 1991, 1993; Ajlani and Vernotte 1998; Domínguez-Martín et al. 2022). Without the linker domains of ApcE, the PB complex is not formed. However, the phycocyanobilin pigment of ApcE can be removed from PB through site-directed mutagenesis, i.e., a substitution of cysteine 190 (covalently binding the phycocyanobilin pigment to the protein matrix; numbering as in *Synechocystis*) with another amino acid residue (Fig. 1) (Gindt et al. 1994; Jallet et al. 2012). Thus, it is possible to deplete the PBs of the excitation energy transfer functions of ApcE but to preserve its structure. Mutants in which cysteine 190 was replaced with a serine in ApcE were previously constructed in *Synechococcus* PCC 7002 (Gindt et al. 1994) or *Synechocystis* (Jallet et al. 2012), and their PBs (ApcE-C190S-PBs) were characterised. For both mutant strains, the bulk fluorescence emission spectra of ApcE-C190S-PBs were blue-shifted when compared to the WT-PBs (Gindt et al. 1994; Jallet et al. 2012). Thus, while only 2 phycocyanobilin pigments are missing in the ApcE-C190S-PBs (leaving a total of up to 394 pigments vs 396 pigments in *Synechocystis’* WT-PB) (Fig. 1), the emission from the pigments in ApcE is, indeed, red-shifted in comparison to other pigments in APCs, and ApcE is one of the TEs receiving excitation energy from the other pigments in the complex (Shen et al. 1993; Gindt et al. 1994; Jallet et al. 2012; van Stokkum et al. 2018). Interestingly, when ApcE-C190S-PBs are isolated in darkness, the pigment binding site of ApcE is still occupied by a phycocyanobilin pigment that is non-covalently bound, emits further to the red, and is photounstable (Gindt et al. 1992, 1994; Jallet et al. 2012; Miao et al. 2016). Upon short pre-illumination of the isolated ApcE-C190S-PBs, the pigment is no longer visible spectroscopically, and this change is irreversible—i.e., the pigment is likely photobleached or leaves the site (Jallet et al. 2012). Such behaviour was only observed in the case of pigments in reengineered PBs.

Single-molecule spectroscopy (SMS) is a powerful tool to study molecular processes taking place in PBs and their subunits (Loos et al. 2004; Goldsmith and Moerner 2010; Long et al. 2015; Wang and Moerner 2015; Squires and Moerner 2017; Gwizdala et al. 2018b, a; Squires et al. 2019; Moya et al. 2022), as well as in other LHCs (for recent reviews see: (Kondo et al. 2017; Gruber et al. 2018)). Our previous SMS studies on isolated PBs revealed a molecular mechanism governing excitation energy flow in PBs that could be rapidly activated in cells exposed to excessive illumination (Gwizdala et al. 2016). This mechanism is intrinsic for PBs (i.e., it does not require the Orange Carotenoid Protein (Kay Holt and Krogmann 1981; Kerfeld et al. 2003, 2017; Wilson et al. 2006; Bao et al. 2017; Gwizdala et al. 2018a; Kirilovsky 2020; Domínguez-Martín et al. 2022)) and involves light-induced switching of pigments between different emissive states. Each pigment in the isolated complex can either be in a *bright* state, which signifies that an excited state lasts long enough to decay by means of fluorescence emission (representing an effective light-harvesting state in vivo), or in a *dark* state (Goldsmith and Moerner 2010; Wang and Moerner 2015), in which the excited-state lifetime is shortened by energy quenching (vide infra for the mechanistic explanation of dark states). A single pigment in a dark state in PB can lead to a significant decrease in the emission intensity and a shortening of the fluorescence lifetime of the whole complex (Krüger et al. 2019). Since the excitation energy transfer in PBs is diffusion-limited, a pigment in a dark state does not instantly quench all the excitation energy in the complex, and, as a result, some emission from the complex still takes place. Thus, a complex with a single pigment in a dark state may be considered to be in a “*dim*” state. The probability of entering such a dim state increases with increasing excitation light intensity (Gwizdala et al. 2016).

Although all pigments of PBs are capable of entering a dark state, energy quenching in an intact PB complex predominantly occurs in the pigments of the core (APC or TEs) (Gwizdala et al. 2016). It remains to be established whether it is solely because an excitation is most likely to be found in these red-shifted compartments of PBs or whether subunits of the core have specific structural arrangements that make their pigments more likely to act as energy quenchers. When a pigment in the core enters a dark state, the fluorescence intensity drastically decreases, the fluorescence lifetime is proportionally shortened, and the emission is blue-shifted. However, if a pigment belonging to a higher-energy compartment of PB, e.g., a PC rod, assumes a dark state, the fluorescence intensity decrease, lifetime shortening, and blue-shifts are less pronounced (i.e., a smaller fraction of excitation energy in the PB complex is quenched), yielding the so-called *intermediate* quenching states (Gwizdala et al. 2016). Already earlier studies showed that the properties of PBs and their subunits can be explored using SMS and that the subunits of PBs display dynamic behaviour, even though PBs photobleach easily (Loos et al. 2004; Goldsmith and Moerner 2010). Various physicochemical mechanisms were suggested to explain these dynamics (Wang and Moerner 2015; Navotnaya et al. 2022); however, a recent Stark spectroscopy study coupled with SMS studies showed that energy quenching in PBs involves light-induced dark states with a charge transfer character (Krüger et al. 2019; Wahadoszamen et al. 2020). Thus, it was proposed that the underpinning mechanism behind the dynamics of PBs emission is related to charge-transfer states.

In this work, we investigate whether ApcE in the core of PBs is a significant player in the rapid light-induced excitation energy quenching mechanism. While our previous studies showed that the core is the most probable site of quenching (Gwizdala et al. 2016; Krüger et al. 2019), at that time, we were unable to point at the specific subunits of the core where the quenching is most likely to take place. Here, we addressed this limitation by exploring the properties of individual ApcE-C190S-PBs from a mutant of *Synechocystis* (Jallet et al. 2012) using SMS and a two-state analysis (Krüger et al. 2011b, a; Valkunas et al. 2012; Schörner et al. 2015; Assefa et al. 2021) and comparing them with the PBs isolated from WT *Synechocystis* (WT-PBs). This study also builds on previous investigations of ApcE-C190S-PBs involving bulk methods (Gindt et al. 1992, 1994; Jallet et al. 2012). The sensitivity of SMS allowed us to identify differences between complexes that differ only by two pigments out of nearly 400. Our study of ApcE-C190S-PBs enhances the understanding of the involvement of different components of PB in the excitation energy flow and regulation in these complexes, which is critical for the success of photosynthesis under fluctuating environmental conditions.

## Results and discussion

### Light-induced PB dynamics

**Fig. 2:**
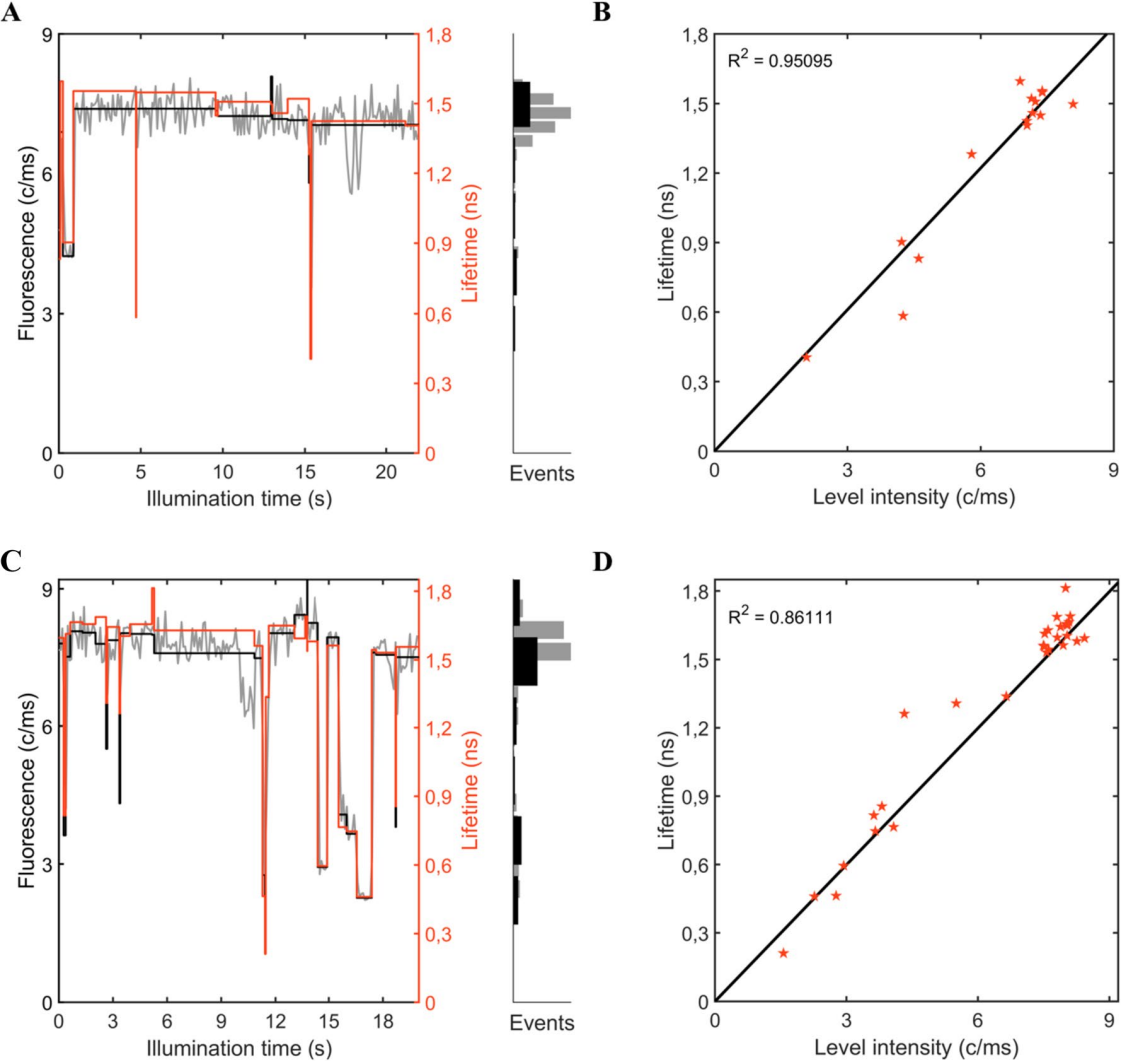
Examples of the dynamic switching of fluorescence intensity and lifetimes, and their correlations for **A** and **B** ApcE-C190S-PB and **C** and **D** WT-PB at 2448 and 2480 mW cm^−2^ illumination, respectively. **A** and (**C** fluorescence intensity traces (grey) with intensity levels (black) and fitted fluorescence lifetimes (red). **B** and **D** linear correlation (black) between measured fluorescence intensity and lifetimes (red stars)

The fluorescence counts from individual WT-PBs and ApcE-C190S-PBs were recorded over a period of tens of seconds using an SMS setup (Fig. 2A and C). For both types of PBs, these measurements revealed significant dynamics in fluorescence intensity and corresponding fluorescence lifetimes. Under continuous excitation, the complexes switched between bright states, characterised by a high number of counts and long fluorescence lifetimes (~ 1,6 ns), and dimmer states of emission, in which the number of counts was decreased and the fluorescence lifetimes were shortened. We will refer to the most strongly quenched states as “*dim states*” and those corresponding to weaker quenching as “*intermediate states*”. As shown previously, energy quenching and dimmer emission states from a PB complex can be explained by a single pigment entering a light-induced dark state and serving as a quencher for the complex (Krüger et al. 2019). The correlation between fluorescence intensity and lifetime of all the emission states (Fig. 2B and D) and the reversibility of the switches between states with different intensities signify that excitation energy quenching in the complexes is responsible for the appearance of the dimmer states (Gwizdala et al. 2016). The lack of two phycocyanobilin pigments in the ApcE subunits of ApcE-C190S-PBs did not change the capacity of these complexes to enter dimmer states and dissipate energy, as judged from a visual inspection of the intensity-lifetime traces.

**Fig. 3:**
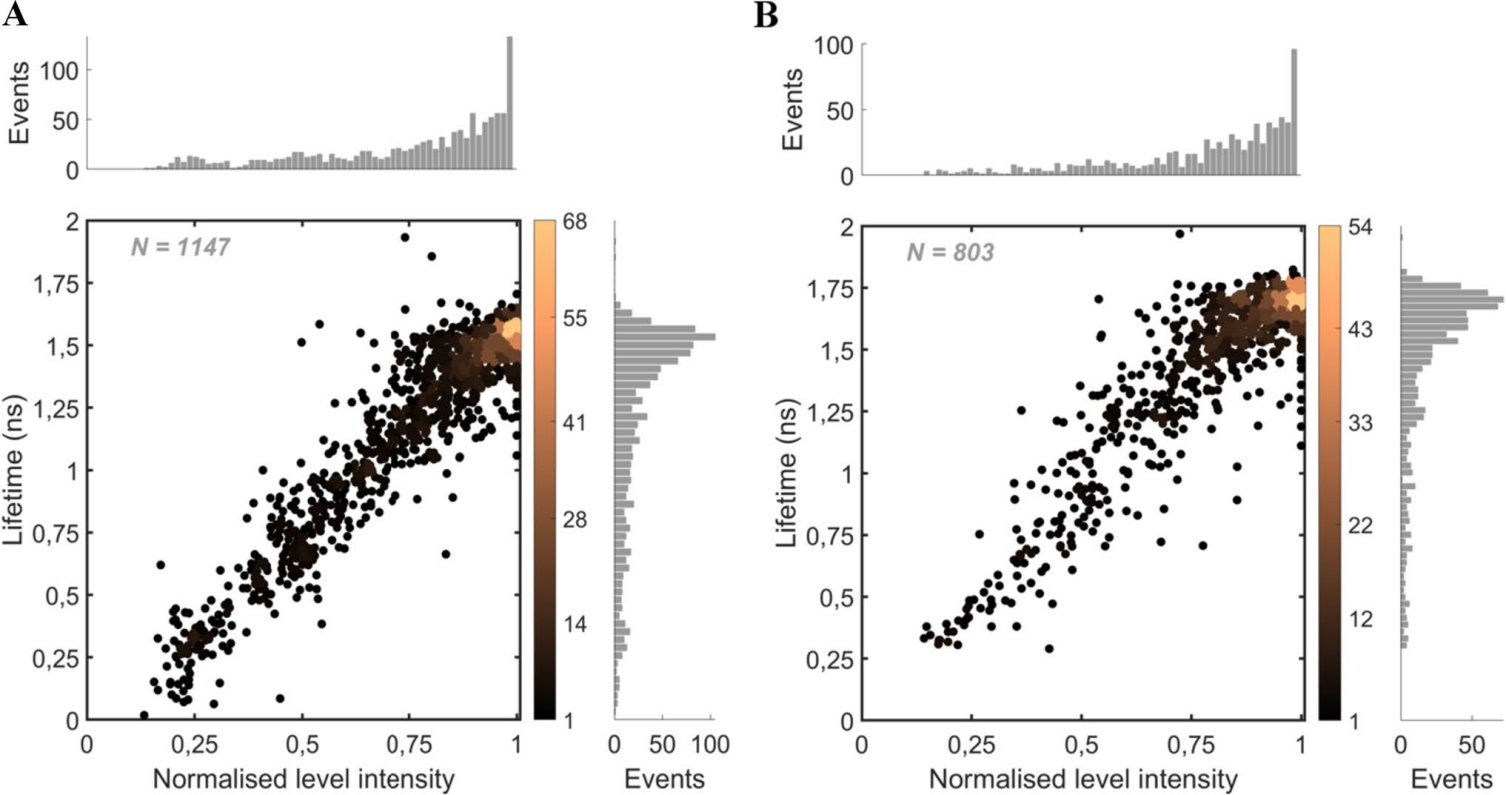
Correlation between the normalised fluorescence intensity and lifetimes for **A** 129 ApcE-C190S-PB and **B** 87 WT-PB complexes at 1648 mW cm^−2^ illumination (Fig. S1 shows the respective non-normalised correlations and Fig. S2 displays the correlation for other light intensities). ***N*** denotes the number of states and the colour bars quantify the number of states per dot area

To compare the fluorescence lifetimes with intensity (Fig. 3), we first pre-screened the datasets to include in the analysis only the complexes that were representative of intact or nearly intact PBs that did not undergo irreversible photodamage during the measurement (see details of pre-screening in the Methods section, and Table S1). The pre-screening ensured that we only considered complexes characterised by a similar absorption cross-section (i.e., similar number of pigments per complex), which allowed us to maintain a constant excitation rate across the selected complexes. These criteria ensure that the conclusions drawn from these experiments are reflective of the properties of PBs as they are in the cells. However, in a previous study, we showed that pre-screening alone does not always remove data from incomplete or photodamaged PBs from the dataset, nor does it correct any setup-related errors such as drift in the excitation beam focus, direction, or intensity, or slight differences in setup alignment between measurement days (Assefa et al. 2021). Thus, in addition to the pre-screening, we also introduced normalisation of the fluorescence intensity to avoid artificial broadening of the intensity distribution to smaller intensity values (compare Fig. 3 with Fig. S1). Without such normalisation, the broadening of the intensity–lifetime distributions may lead to an incorrect interpretation, such as the appearance of a new type of state characterised by low fluorescence intensity and long fluorescence lifetime.

The correlation between normalised fluorescence intensity and fluorescence lifetimes for the pre-screened complexes showed that both ApcE-C190S-PBs and WT-PBs typically switched between a bright state and a quasi-continuum of dimmer states (Fig. 3A and B). For a range of excitation light intensities (Fig S2 and S3 for ApcE-C190S-PBs and WT-PBs), the bright state is characterised by average fluorescence lifetimes of 1,65 ± 0,01 ns and 1,55 ± 0,02 ns for WT-PBs and ApcE-C190S-PBs, respectively. The shorter lifetime of ApcE-C190S-PB in its bright state could be explained by the ApcE subunit having a longer fluorescence lifetime than the other subunits as hesitantly suggested by (Long et al. 2015). However, this longer lifetime of ApcE remains to be confirmed.

**Fig. 4:**
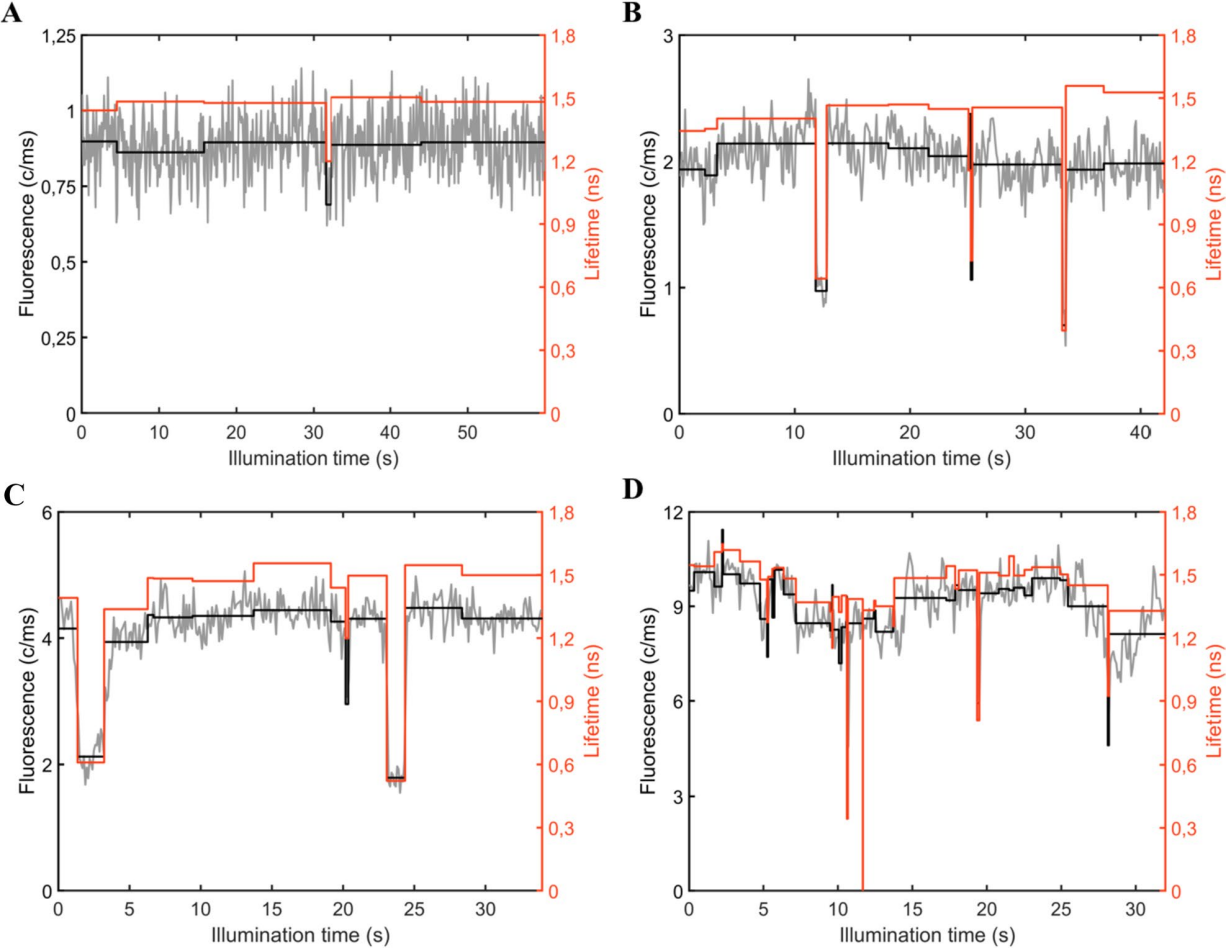
Examples of increasing emission and lifetime dynamics for the ApcE-C190S-PB with increasing excitation rates: **A** 400, **B** 896, **C** 1648, and **D** 4096 mW cm^−2^. The fluorescence intensity traces (grey) with intensity levels (black) and fitted fluorescence lifetimes (red). Fig. S4 shows analogous examples for WT-PB

The quasi-continuum of intermediately quenched and dim states suggests that energy quenching can occur in any part of the complex. Deeply quenched (dim) states in Fig. 3A and B (i.e., those signified by the shortest fluorescence lifetimes and the lowest intensity) are most likely related to quenching in the low-energy compartments of PBs, i.e., the core, because the majority of excitations are affected by quenching in the core (Gwizdala et al. 2016; Krüger et al. 2019). The intermediate states, on the other hand, are likely due to quenching in the distal, high-energy parts of the PBs (e.g., PC rods), because when quenching takes place in one of the rods, excitation energy transfer from the other rods to the core is unaffected (van Stokkum et al. 2018). Thus, the distinction between the deeply quenched, dim states, and intermediate states derives from and is related to the hand-like structure of hemi-discoidal PBs and the unidirectionality of energy transfer in these complexes. An alternative explanation for the intermediate states could be related to various quenching strengths of pigments in the dark states. However, a previous study on APC trimers indicated the presence of only one type of dark state in these complexes (Wang and Moerner 2015). A comparison of the intensity state distribution for ApcE-C190S-PBs and WT-PBs in Fig. 3A and B does not reveal any significant differences between the two types of complexes as their global structure and number of pigments remain similar. We have previously shown that the magnitude of switching between different emission states in WT-PBs changes with the excitation light intensity (Gwizdala et al. 2016). A similar response to illumination was observed for ApcE-C190S-PBs. With increasing excitation rate—within the range 423 × 10^3 ^– 4423 × 10^3^ and 294 × 10^3 ^– 4458 × 10^3^ photons absorbed per complex per second for ApcE-C190S-PBs and WT-PBs, respectively—the complexes switched more frequently between different states as shown in representative examples in Fig. 4 for ApcE-C190S-PBs and Fig. S4 for WT-PB. It is worth pointing out that even at the highest excitation rate used in this study, the probability of singlet–singlet annihilation was 9,3 × 10^–4^ and thus negligible.

### Multistep model quantification of PBs dynamics

**Fig. 5:**
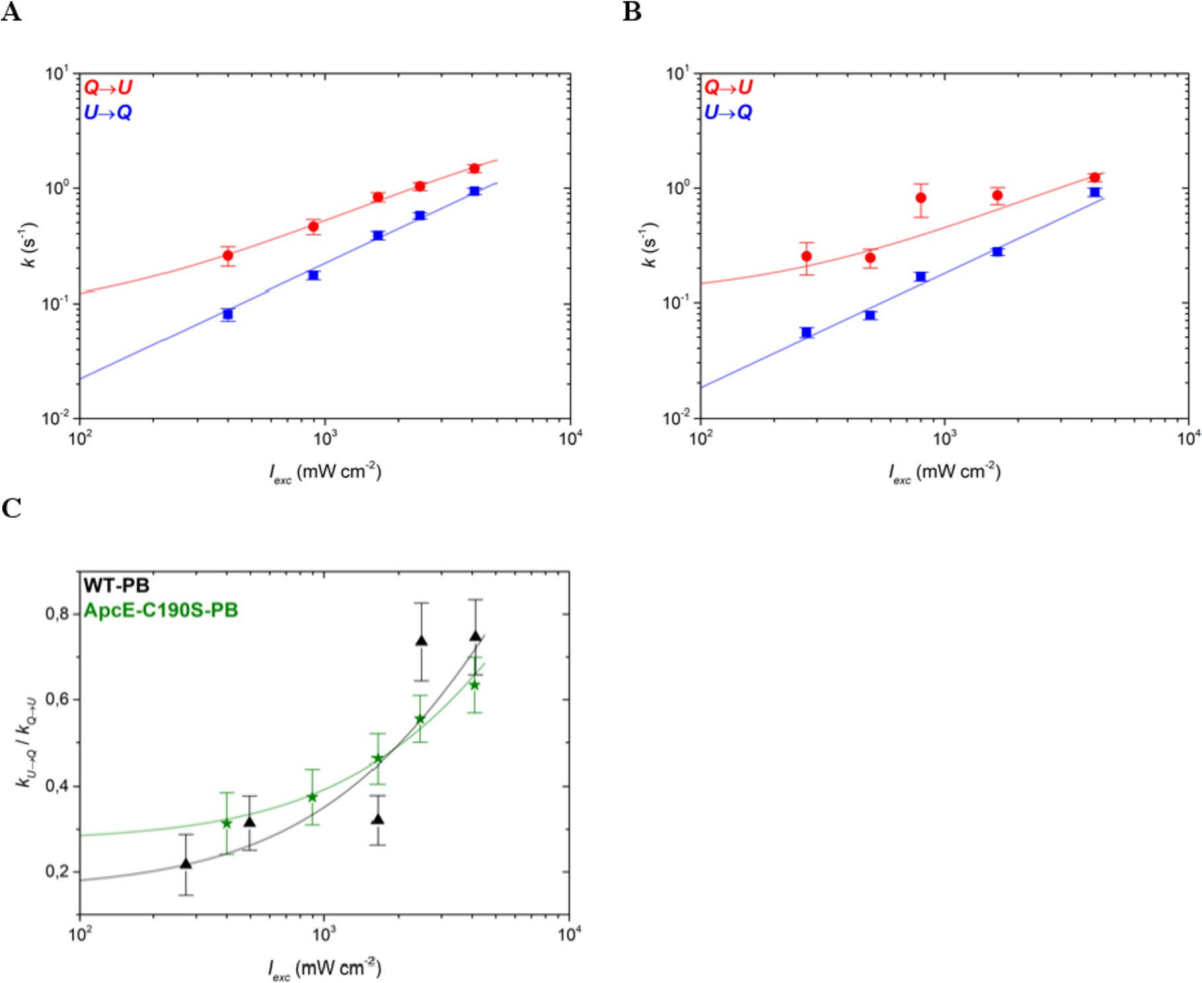
Switching rates following a multistate model for **A** ApcE-C190S-PBs and **B** WT-PBs, and **C** the ratio of the two switching rates for both types of complexes for ApcE-C190S-PB (green stars) and WT-PB (black triangles). In **A** and **B**, switches from quenched to unquenched states (*Q* → *U*) are shown as red circles and switches in the opposite direction (*U* → *Q*) as blue squares. The latter were fitted with linear regression lines (blue), while the former were fitted with logarithmic functions derived from Gwizdala et al. 2016 (red lines). Fits in **C** serve to guide the eye. Error bars denote standard errors

Since the complexes switched between a large number of different intensity states, we first used a multistep analysis model to sample the global fluorescence intensity dynamics of the complexes, the same approach that was used in an earlier study (Gwizdala et al. 2016). In this approach, every statistically significant change in the fluorescence intensity between two consecutive intensity levels counts as an intensity switch. The switch can be to a lower intensity, i.e., from a relatively unquenched to a more quenched state (*U → Q*), or a higher intensity level, i.e., from a relatively quenched to a more unquenched state (*Q → U*). Such a multistep analysis includes all the emission dynamics and does not discriminate between dim, intermediate, or strongly emissive (bright) states. Thus, in a multistep analysis, energy quenching in any part of the complex is considered. Applying this approach to the time-resolved SMS data of ApcE-C190S-PBs and WT-PBs allowed us to quantify the switching rates between different states (Fig. 5) and to obtain a global picture of the intensity dynamics. For both types of PBs, the switching rates in both directions (*k*_*U→Q*_ and *k*_*Q→U*_) increased with an increasing excitation rate. More importantly, the ratio of the two rates, *k*_*U→Q*_/ *k*_*Q→U*_, increased with enhanced illumination for both ApcE-C190S-PBs and WT-PBs (Fig. 5C), signifying that both complexes respond to enhanced illumination by shifting their dynamic intensity equilibrium towards quenched states, the essence of the intrinsic light-induced quenching mechanism (Gwizdala et al. 2016). In other words, with increasing light intensity, the switching rates towards the quenched states (*k*_*U→Q*_) grow faster than the switching rates towards the unquenched states (*k*_*Q→U*_) and, on average, the complexes dissipate a larger fraction of excitations. Since the switching rate ratios of WT-PBs and ApcE-C190S-PBs are mostly similar within the error margins (Fig. 5C), the removal of the two pigments from the ApcE subunits of ApcE-C190S-PBs had a negligible effect on the overall free energy landscape of the complex. This result is not unexpected since the two types of complexes in this study should have similar overall structures and differ by only two phycocyanobilin pigments (out of 396) and each intensity decrease likely corresponds to quenching at the level of a single pigment (Krüger et al. 2019).

### Two-state model analysis of PB dynamics

An alternative, equally simple but complementary approach to the switching analysis involves a two-state model that highlights the processes taking place exclusively in the PBs core as opposed to the multistep model describing the global dynamics of complexes (Assefa et al. 2021). Due to a large number of pigments in PBs – each capable of becoming a quencher when excited – the distribution of states in PBs seems quasi-continuous (e.g., Fig. 3). However, by assuming a coarse structural model of a PB – composed of the rods and the core – and knowing that the strongest quenching takes place in the core (Gwizdala et al. 2016), the two-state model gives access to the emission dynamics of the core. It is worth noting that a two-state model was similarly used to focus on large intensity changes attributed to the terminal-emitter chlorophyll *a* cluster in the main plant light-harvesting complex LHCII (Krüger et al. 2012).

By considering that at a given time, a complex can either be in a bright or a dim state separated by a threshold, a two-state analysis only accounts for switches across the threshold, i.e., those involving energy quenching in the PBs core. Energy quenching in the rods is not expected in this analysis model because a switch from a bright to an intermediate state typically does not involve crossing the threshold. Here, the threshold positions were determined as a saddle point between the Gaussians fitted to the fluorescence lifetime distributions (Fig. S5 and S6 and Table S2) and had average values of 1,23 ± 0,10 ns and 1,29 ± 0,07 ns for the ApcE-C190S-PBs and WT-PBs, respectively (standard error as uncertainty). These Gaussians compare well with the distributions of the bright (unquenched) and dim (quenched) states resolved from a three-state Gaussian mixture model (Fig. S7) and highlight again that large-intensity changes correspond to switches between these bright and dim states. It is worth noticing that for a given type of PB there are no significant differences in the peak position of fitted Gaussians across different excitations (Table S2 and Fig. S8) and the threshold positions also remain significantly unchanged (Table S2), justifying our fitting procedure. We have previously demonstrated that the threshold position has little impact on the two-state analysis, especially for stable samples (Assefa et al. 2021), further justifying the complex preselection procedure used in this study to only analyse intact complexes.

**Fig. 6:**
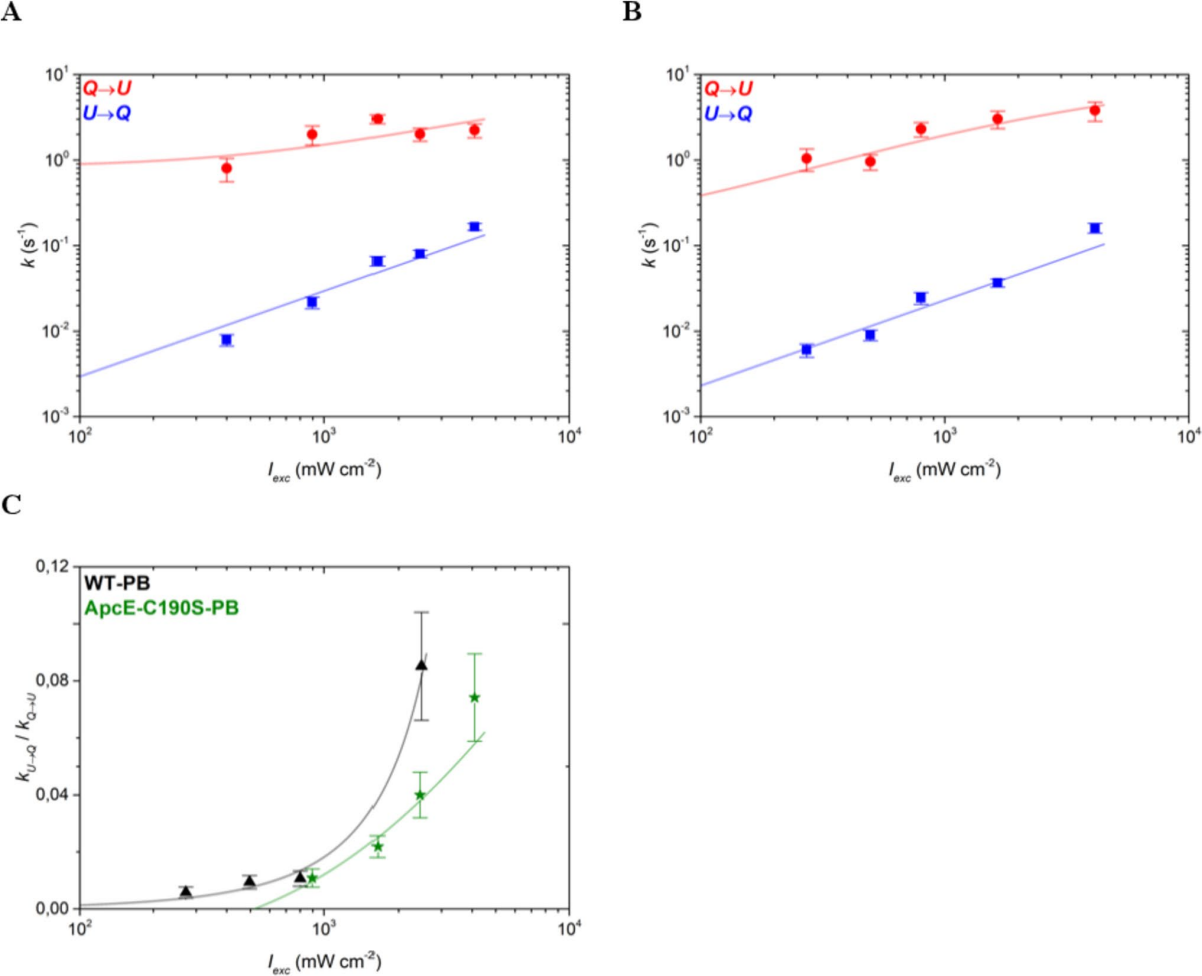
Switching rates following a two-state model for **A** ApcE-C190S-PBs and **B** WT-PBs, and **C** the ratio of the switching rates ratios for both types of complexes for ApcE-C190S-PB (green stars) and WT-PB (black triangles). In **A** and **B**, switches from quenched to unquenched states (*Q* → *U*) are shown as red circles and switches in the opposite direction (*U* → *Q*) as blue squares. Regression fits were performed as described for Fig. 5. Error bars denote standard errors

The general trend in the two-state analysis is similar to the multistate model and shows that the switching rates ${k}_{U\to Q}$ and ${k}_{Q\to U}$ both increase with an enhanced excitation rate (Fig. 6A and B), indicating that with increasing light intensity, the PB cores become more dynamic. Importantly, the ratio ${k}_{U\to Q}/{k}_{Q\to U}$ revealed a striking difference between WT-PBs and ApcE-C190S-PBs (Fig. 6C). While the general trends are again similar, i.e., the ratio of switching rates increases with the excitation intensity, the values for ApcE-C190S-PBs are significantly lower than for WT-PBs across the range of investigated light intensities. Thus, ApcE-C190S-PBs respond less to illumination than WT-PBs. This difference is significant, considering that it results from the effect of the removal of only two out of almost 400 pigments. It demonstrates that the pigments of ApcE play an important role in light-induced energy dissipation in PBs. While the lack of pigments in ApcE hinders the response of the PB core to light it does not completely block it, confirming that other subunits of the core also play a role in light-induced energy dissipation.

### Spectral analysis

**Fig. 7:**
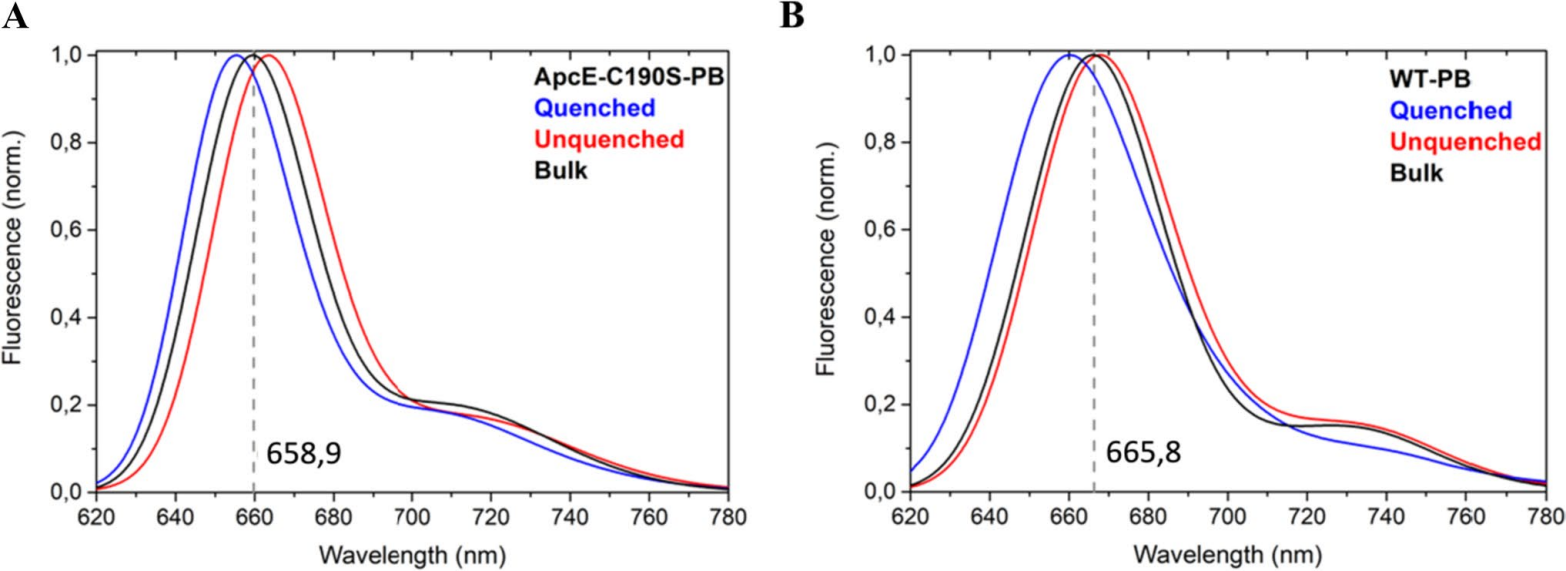
Emission spectra of PBs in unquenched (red) and strongly quenched (blue) states (according to the two-state model analysis) compared with the bulk (black) spectra of **A** ApcE-C190S-PBs and **B** WT-PBs. Grey dashed line shows the peak position of the bulk spectra. All spectra were collected using the same optical setup

Maintaining the threshold from the two-state analysis, we next compared the fluorescence emission spectra of ApcE-C190S-PBs and WT-PBs in bright and dim states with the bulk spectrum (Fig. 7). The bulk fluorescence emission spectra of ApcE-C190S-PBs are blue-shifted by 6,9 nm compared to WT-PBs due to the missing pigment of the red-shifted ApcE subunit, in agreement with previous reports (Jallet et al. 2012). For each type of PB in this study, the fluorescence emission spectra of complexes in dim states were blue-shifted from the bulk spectrum, while the spectra of complexes in bright states were somewhat red-shifted (Fig. 7). In addition, the ApcE-C190S-PBs were capable of entering reversible far-red emission states, similar to the states described previously for WT-PBs (Fig S9) (Gwizdala et al. 2016; Krüger et al. 2019; Wahadoszamen et al. 2020). These observations demonstrate that the bulk spectra constitute averages over the complexes’ full spectral heterogeneity that is accessible through SMS, i.e., at any given moment, the bulk contains subpopulations of complexes in bright states, dim states, and far-red emission states, respectively. Moreover, the average spectra of all individually measured ApcE-C190S-PBs in bright or dim states were blue-shifted in comparison to the respective WT-PBs average spectra. The far-red states indicate that the physicochemical origins of energy quenching are the same in ApcE-C190S-PBs and WT-PBs and involve charge-transfer states (Krüger et al. 2019; Wahadoszamen et al. 2020). Since the complexes were briefly illuminated during the raster scan before the onset of the spectral measurements, we did not observe the spectroscopic signatures of the non-covalently ApcE-bound phycocyanobilin pigment in ApcE-C190S-PBs. Like for ensemble measurements, these signatures would irreversibly disappear upon initial illumination (Jallet et al. 2012), which, in the case of the SMS experiments occurred before data collection.

Importantly, the average single-molecule spectra for both types of PBs investigated in this study overlap well with the bulk spectra (Fig S10). Their peak positions as well as the full-width half maxima are nearly identical (Table S3), indicating that the single-molecule conditions used in this study did not noticeably impact the complexes.

## Conclusions

While it is impossible to study PBs deprived of their ApcE subunits, it was possible to use SMS to explore the properties of ApcE-C190S-PB complexes depleted of the ApcE pigments. Despite the crucial role of ApcE in excitation energy transfer to the RCs, the lack of its pigment did not introduce significant global spectroscopic changes in isolated PBs other than a blue shift in their emission spectra and a slight shortening of fluorescence lifetime. However, a two-state intensity data analysis model, which provides information about the emission dynamics in the PB core, clearly demonstrated that in the case when the ApcE pigments are not involved in excitation energy transfer, PBs are less capable of entering states in which excitation energy is dissipated, thus potentially affecting their ability to switch into a photoprotective state. We conclude that ApcE is not only responsible for feeding photosynthetic RCs with excitations captured by the PBs, but also plays an important role in the regulation of light-induced excitation energy flow in PBs.

## Methods

### Sample preparation

The WT-PB and C190S-ApcE-PB were isolated from WT *Synechocystis* PCC6803 and from a mutant (Jallet et al. 2012) as described in (Gwizdala et al. 2011). Samples were thawed 30 min before the measurement and diluted ~ 10^6^ times with 0,8 M K-phosphate buffer at pH 7,5 directly before the measurement.

### SMS measurements

SMS measurements were performed at room temperature in the presence of air following the protocols described in (Gwizdala et al. 2016), but using a different custom-built SMS setup, described previously in (Kyeyune et al. 2019). The setup was modified to measure fluorescence emission from PBs (see the spectra of the optical filters given in Fig. S11) according to (Gwizdala et al. 2016). Briefly, pulsed 594 nm laser light (Fianium Supercontinuum laser (SC400-4-PP)) excited the PBs immobilized on poly-L-lysine (PLL)-coated coverslips. The same objective (Nikon, 1.45 NA, oil immersion) was used for excitation and emission. Fluorescence passed through a dichroic beam splitter (605dcxt, Chroma Technology Corp.) and fluorescence filters (600LPF, Edmund optics, and KC-13, OÜ Maico Metrics) before being projected onto the detectors. Using a non-polarising beam splitter, 70% of the fluorescence was directed onto a single-photon avalanche photodiode (Micro Photon Devices PD-050-CTE or Excelitas SPCM-AQR-16) coupled to a time-correlated single-photon counting (TCSPC; SPC-134, Becker & Hickl GmbH) unit used to register time-tagged time-correlated photons, which were subsequently used to construct fluorescence intensity traces and corresponding lifetime histograms. Fluorescence emission spectra (30% of the fluorescence) were collected with an Electron Multiplying Charge-Coupled Device (EMCCD; Andor iXon3) camera after dispersing the light by a diffraction grating (Thorlabs, GR25–0608, 600 grooves/mm, 750 nm blaze). All measurements were performed at room temperature and in the presence of oxygen. Bulk spectra were collected using the same SMS setup.

### Data pre-selection

A modified pre-selection procedure from (Gwizdala et al. 2016) was used in this study. Briefly, all intensity traces were first fitted with intensity levels using a home-written script employing the change-point algorithm of (Watkins and Yang 2005). For levels binning over 500 photons, the fluorescence lifetimes were resolved using a home-written script adapting the method described in (Smith et al. 2017). Fluorescence lifetime-intensity correlations were then established for each complex individually and the distribution of the slopes of these correlations – as indicators of the relative size of the complexes – was fitted with a Gaussian function. Only complexes whose slope was within 2 standard deviations from the average and whose maximal fluorescence lifetimes were between 1,5 and 2 ns were included. To eliminate setup-related variations in fluorescence intensity or any possible variations related to sample orientation on the microscope cover glass, the intensity level values were proportionally normalised to 100% for the level of highest counts for each pre-selected complex as in (Assefa et al. 2021).

### Data analysis

The threshold position in the two-state analysis was established as the local minimum between two Gaussians fitted to the distribution of fluorescence lifetimes. For these fits, initially, half-Gaussians were fitted to the outer edges of the distributions and then mirroring second halves were added to complete both Gaussians. The threshold was then used to divide states assumed by the complexes as bright, “unquenched” states (characterised by lifetimes longer than or equal to the threshold value) and dim, “quenched” states (with lifetimes shorter than the threshold value). The multistate analysis was performed as in (Gwizdala et al. 2016) and the switching rates ${k}_{U\to Q}$ and ${k}_{Q\to U}$ were defined as follows:

$$k_{U \to Q} = \frac{{N\left( {U \to Q} \right)}}{{\sum \tau_{U} }}\,{\text{and}}\,k_{Q \to U} = \frac{{N\left( {Q \to U} \right)}}{{\sum \tau_{Q} }}$$

where *N* is the total number of resolved switches in a given direction, and *τ* denotes the total dwell time in unquenched (*τ*_U_) or quenched states (*τ*_Q_).

Fluorescence lifetimes were resolved for all intensity levels binning over 500 photons, by deconvolving the fluorescence lifetime distribution histogram with a monoexponential function and the measured instrument response function (IRF) of the setup (Fig. S12). The IRF was measured by using back-scattered light at 665 nm that passed through all optical components of the setup.

Fluorescence emission spectra were fitted with Gaussians as described previously (Gwizdala et al. 2016). Error bars shown in the figures are standard errors. The analysis was performed using in-house written Python, Matlab, or Origin 9.1 codes and isualised using PyMol 1.6 and Origin 9.1.

### Supplementary Information

Below is the link to the electronic supplementary material.

Supplementary file (PDF 2754 KB)

## Data Availability

Experimental data is available upon request.

## References

[CR1] Adir N (2005). Elucidation of the molecular structures of components of the phycobilisome: reconstructing a giant. Photosynth Res.

[CR2] Ajlani G, Vernotte C (1998). Deletion of the PB-loop in the L-CM subunit does not affect phycobilisome assembly or energy transfer functions in the cyanobacterium synechocystis sp. PCC6714. Eur J Biochem.

[CR3] Arteni A, Ajlani G, Boekema E (2009). Structural organisation of phycobilisomes from Synechocystis sp strain PCC6803 and their interaction with the membrane. Biochim Biophys Acta, Bioenerg.

[CR4] Ashby M, Mullineaux C (1999). The role of ApcD and ApcF in energy transfer from phycobilisomes to PSI and PSII in a cyanobacterium. Photosynth Res.

[CR5] Assefa GT, Krüger TPJ, Gwizdala M (2021). Impact of the intensity threshold on binary switching analysis in single molecule spectroscopy of phycobilisomes. In: Single Molecule Spectroscopy and Superresolution Imaging XIV..

[CR6] Bao H, Melnicki MR, Kerfeld CA (2017). Structure and functions of orange carotenoid protein homologs in cyanobacteria. Curr Opin Plant Biol.

[CR7] Calzadilla PI, Muzzopappa F, Sétif P, Kirilovsky D (2019). Different roles for ApcD and ApcF in synechococcus elongatus and synechocystis sp. PCC 6803 phycobilisomes. Biochim Biophys Acta, Bioenerg.

[CR8] Capuano V, Thomas JC, Tandeau de Marsac N, Houmard J (1993). An in vivo approach to define the role of the LCM, the key polypeptide of cyanobacterial phycobilisomes. J Biol Chem.

[CR9] Capuano V, Braux A, Tandeau de Marsac N, Houmard J (1991) The “anchor polypeptide” of cyanobacterial phycobilisomes. Molecular characterization of the Synechococcus sp. PCC 6301 apce gene. http://www.jbc.org/content/266/11/7239.full.pdf+html?frame=sidebar. Accessed 6 Jan 2010 1901865

[CR10] de Marsac NT, Cohen-bazire G (1977). Molecular composition of cyanobacterial phycobilisomes. Proc Natl Acad Sci U S A.

[CR11] Domínguez-Martín MA, Sauer PV, Kirst H (2022). Structures of a phycobilisome in light-harvesting and photoprotected states. Nature.

[CR12] Duerring M, Schmidt GB, Huber R (1991). Isolation, crystallization, crystal structure analysis and refinement of constitutive C-phycocyanin from the chromatically adapting cyanobacterium Fremyella diplosiphon at 1.66 Å resolution. J Mol Biol.

[CR13] Gindt Y, Zhou J, Bryant D, Sauer K (1992). Core mutations of synechococcus Sp PCC-7002 phycobilisomes—a spectroscopic study. Journal of Photochemistry and Photobiology B-Biology.

[CR14] Gindt Y, Zhou J, Bryant D, Sauer K (1994). Spectroscopic studies of phycobilisome subcore preparations lacking key core chromophores—assignment of excited-state energies to the L(Cm), Beta(18) and Alpha(Ap-B) Chromophores. Biochim Biophys Acta, Bioenerg.

[CR15] Glazer AN (1984). Phycobilisome a macromolecular complex optimized for light energy transfer. Biochim Biophys Acta.

[CR16] Goldsmith RH, Moerner WE (2010). Watching conformational- and photodynamics of single fluorescent proteins in solution. Nat Chem.

[CR17] Gruber JM, Malý P, Krüger TPJ, van Grondelle R (2018). From isolated light-harvesting complexes to the thylakoid membrane: a single-molecule perspective. Nanophotonics.

[CR18] Gwizdala M, Wilson A, Kirilovsky D (2011). In Vitro reconstitution of the cyanobacterial photoprotective mechanism mediated by the orange carotenoid protein in synechocystis PCC 6803. Plant Cell.

[CR19] Gwizdala M, Berera R, Kirilovsky D (2016). Controlling light harvesting with light. J Am Chem Soc.

[CR20] Gwizdala M, Botha JL, Wilson A (2018). Switching an individual phycobilisome off and on. J Phys Chem Lett.

[CR21] Gwizdala M, Krüger TPJ, Wahadoszamen Md (2018). Phycocyanin: one complex, two states, two functions. J Phys Chem Lett.

[CR22] Jallet D, Gwizdala M, Kirilovsky D (2012). ApcD, ApcF and ApcE are not required for the orange carotenoid protein related phycobilisome fluorescence quenching in the cyanobacterium synechocystis PCC 6803. Biochim Biophys Acta, Bioenerg.

[CR23] Kay Holt T, Krogmann DW (1981). A carotenoid-protein from cyanobacteria. Biochim Biophys Acta, Bioenerg.

[CR24] Kerfeld CA, Sawaya MR, Brahmandam V (2003). The crystal structure of a cyanobacterial water-soluble carotenoid binding protein. Structure.

[CR25] Kerfeld CA, Melnicki MR, Sutter M, Dominguez-Martin MA (2017). Structure, function and evolution of the cyanobacterial orange carotenoid protein and its homologs. New Phytol.

[CR26] Kirilovsky D, Larkum AWD, Grossman AR, Raven JA (2020). Modulating Energy Transfer from Phycobilisomes to Photosystems: State Transitions and OCP-Related Non-Photochemical Quenching. Photosynthesis in Algae: Biochemical and Physiological Mechanisms.

[CR27] Kondo T, Chen WJ, Schlau-Cohen GS (2017). Single-molecule fluorescence spectroscopy of photosynthetic systems. Chem Rev.

[CR28] Krüger TPJ, Ilioaia C, Valkunas L, van Grondelle R (2011). Fluorescence intermittency from the main plant light-harvesting complex: sensitivity to the local environment. J Phys Chem B.

[CR29] Krüger TPJ, Ilioaia C, van Grondelle R (2011). Fluorescence intermittency from the main plant light-harvesting complex: resolving shifts between intensity levels. J Phys Chem B.

[CR30] Krüger TPJ, Ilioaia C, Johnson MP (2012). Controlled disorder in plant light-harvesting complex II explains its photoprotective role. Biophys J.

[CR31] Krüger TPJ, van Grondelle R, Gwizdala M (2019). The role of far-red spectral states in the energy regulation of phycobilisomes. Biochim Biophys Acta, Bioenerg.

[CR32] Kyeyune F, Botha JL, van Heerden B (2019). Strong plasmonic fluorescence enhancement of individual plant light-harvesting complexes. Nanoscale.

[CR33] Liu H, Zhang MM, Weisz DA (2021). Structure of cyanobacterial phycobilisome core revealed by structural modeling and chemical cross-linking. Sci Advances.

[CR34] Long S, Zhou M, Tang K (2015). Single-molecule spectroscopy and femtosecond transient absorption studies on the excitation energy transfer process in ApcE(1–240) dimers. Phys Chem Chem Phys.

[CR35] Loos D, Cotlet M, De Schryver F (2004). Single-molecule spectroscopy selectively probes donor and acceptor chromophores in the phycobiliprotein allophycocyanin. Biophys J.

[CR36] Lundell DJ, Glazer AN (1983). Molecular architecture of a light-harvesting antenna. Structure of the 18 S core-rod subassembly of the Synechococcus 6301 phycobilisome. J Biol Chem.

[CR37] Lundell DJ, Glazer AN (1983). Molecular architecture of a light-harvesting antenna. Core substructure in synechococcus 6301 phycobilisomes: two new allophycocyanin and allophycocyanin B complexes. J Biol Chem.

[CR38] Lundell DJ, Williams RC, Glazer AN (1981). Molecular architecture of a light-harvesting antenna. In vitro assembly of the rod substructures of synechococcus 6301 PHYCOBILISOMES. J Biol Chem.

[CR39] Ma J, You X, Sun S (2020). Structural basis of energy transfer in *Porphyridium* purpureum phycobilisome. Nature.

[CR40] Maxson P, Sauer K, Zhou JH (1989). Spectroscopic studies of cyanobacterial phycobilisomes lacking core polypeptides. Biochim Biophys Acta.

[CR41] Miao D, Ding W-L, Zhao B-Q (2016). Adapting photosynthesis to the near-infrared: non-covalent binding of phycocyanobilin provides an extreme spectral red-shift to phycobilisome core-membrane linker from *Synechococcus* sp. PCC733. Biochim Biophys Acta, Bioenerg..

[CR42] Moya R, Norris AC, Kondo T, Schlau-Cohen GS (2022). Observation of robust energy transfer in the photosynthetic protein allophycocyanin using single-molecule pump–probe spectroscopy. Nat Chem.

[CR43] Navotnaya P, Sohoni S, Lloyd LT (2022). Annihilation of excess excitations along phycocyanin rods precedes downhill flow to allophycocyanin cores in the phycobilisome of synechococcus elongatus PCC 7942. J Phys Chem B.

[CR44] Schörner M, Beyer SR, Southall J (2015). Multi-Level, multi time-scale fluorescence intermittency of photosynthetic lh2 complexes: a precursor of non-photochemical quenching?. J Phys Chem B.

[CR45] Shen G, Boussiba S, Vermaas WF (1993). Synechocystis sp PCC 6803 strains lacking photosystem I and phycobilisome function. Plant Cell.

[CR46] Smith DA, McKenzie G, Jones AC, Smith TA (2017). Analysis of time-correlated single photon counting data: a comparative evaluation of deterministic and probabilistic approaches. Methods Appl Fluoresc.

[CR47] Squires AH, Moerner WE (2017). Direct single-molecule measurements of phycocyanobilin photophysics in monomeric C-phycocyanin. Proc Natl Acad Sci U S A.

[CR48] Squires AH, Dahlberg PD, Liu H (2019). Single-molecule trapping and spectroscopy reveals photophysical heterogeneity of phycobilisomes quenched by orange carotenoid protein. Nat Commun.

[CR49] Valkunas L, Chmeliov J, Krüger TPJ (2012). How photosynthetic proteins switch. J Phys Chem Lett.

[CR50] van Stokkum IHM, Gwizdala M, Tian L (2018). A functional compartmental model of the *Synechocystis* PCC 6803 phycobilisome. Photosynth Res.

[CR51] Wahadoszamen Md, Krüger TPJ, Ara AM (2020). Charge transfer states in phycobilisomes. Biochim Biophys Acta, Bioenerg.

[CR52] Wang Q, Moerner WE (2015). Dissecting pigment architecture of individual photosynthetic antenna complexes in solution. Proc Natl Acad Sci U S A.

[CR53] Watkins LP, Yang H (2005). Detection of intensity change points in time-resolved single-molecule measurements. J Phys Chem B.

[CR54] Wilson A, Ajlani G, Verbavatz J (2006). A soluble carotenoid protein involved in phycobilisome-related energy dissipation in cyanobacteria. Plant Cell.

[CR55] Yamanaka G, Glazer AN, Williams RC (1980). Molecular architecture of a light-harvesting antenna. Comparison of wild type and mutant *Synechococcus* 6301 phycobilisomes. J Biol Chem.

[CR56] Yu MH, Glazer AN, Williams RC (1981). Cyanobacterial phycobilisomes. Phycocyanin assembly in the rod substructures of anabaena variabilis phycobilisomes. J Biol Chem.

[CR57] Zhang J, Ma J, Liu D (2017). Structure of phycobilisome from the red alga *Griffithsia pacifica*. Nature.

[CR58] Zhao K-H, Porra RJ, Scheer H (2012). Phycobiliproteins. Handbook of Porphyrin Science.

[CR59] Zheng L, Zheng Z, Li X (2021). Structural insight into the mechanism of energy transfer in cyanobacterial phycobilisomes. Nat Commun.

[CR60] Zilinskas BA (1982). Isolation and characterization of the central component of the phycobilisome core of nostoc sp. 1 2. Plant Physiol.

