# Supplementary material for: ApcE plays an important role in light-induced excitation energy dissipation in the Synechocystis PCC6803 phycobilisomes
Source: Photosynth Res. 2024 Feb 26;160(1):17–29. doi: 10.1007/s11120-024-01078-6 (PMC11006782; doi:10.1007/s11120-024-01078-6)
Supplement: Supplementary file 1 — Supplementary file (PDF 2754 KB) [file 11120_2024_1078_MOESM1_ESM.pdf]

Supplementary Information
(SI)

ApcE plays an important role in light-induced excitation energy dissipation in the Synechocystis PCC6803 phycobilisomes

Gonfa Tesfaye Assefa, Joshua L. Botha, Bertus van Heerden, Farooq Kyeyune, Tjaart P.J. Krüger, and Michal Gwizdala

Fig. S1

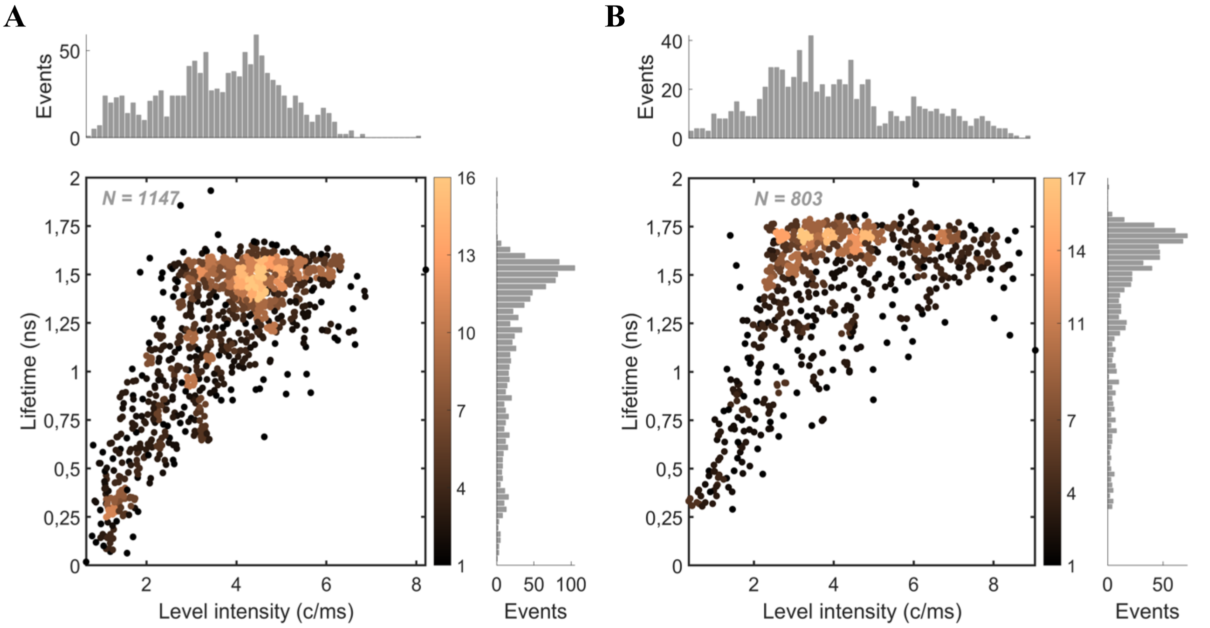

Fig. S1. Correlation between the non-normalised fluorescence intensities and lifetimes for **A)** 129 ApcE-C190S-PB and **B)** 87 WT-PB complexes at 1648 mW cm^{-2} illumination. ***N*** denotes the number of states, and the colour bars quantify the number of states per dot area.

Fig. S2

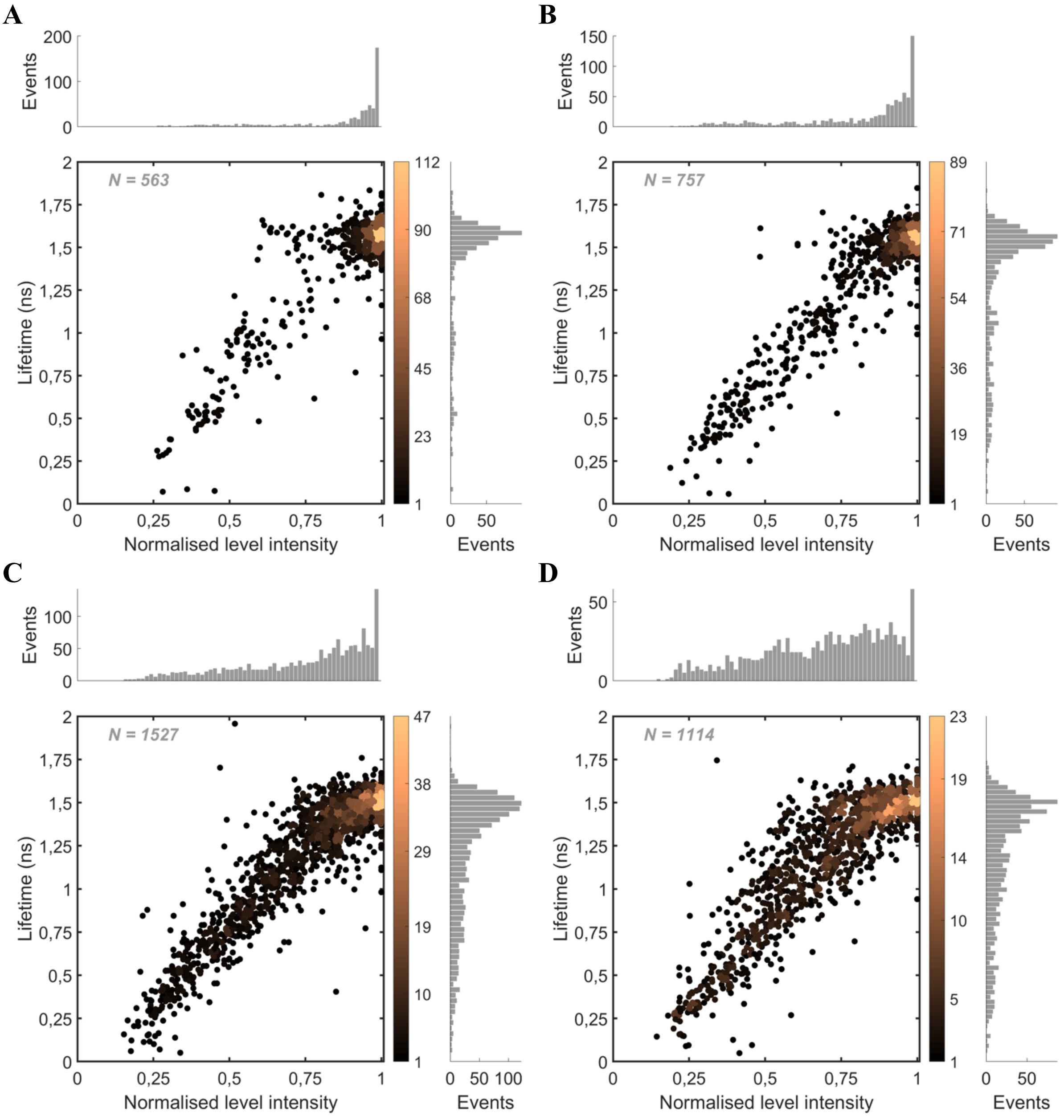

Fig. S2. Correlation between the normalised fluorescence intensities and lifetimes for ApcE-C190S-PB complexes at **A)** 400, **B)** 896, **C)** 2448, and **D)** 4096 mW cm^{-2} illumination. ***N*** denotes the number of states, and the colour bars give the number of states per dot area.

Fig. S3

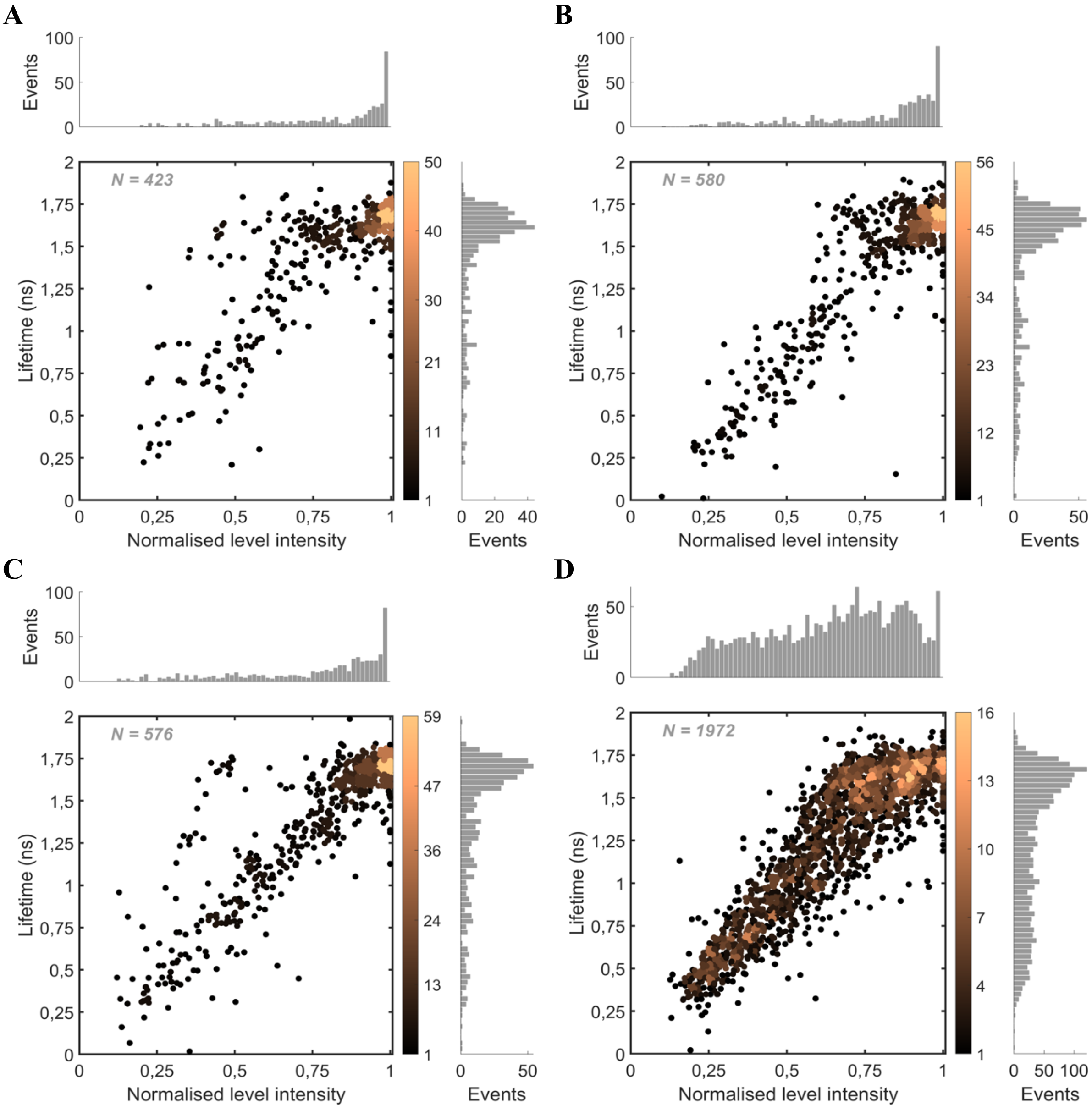

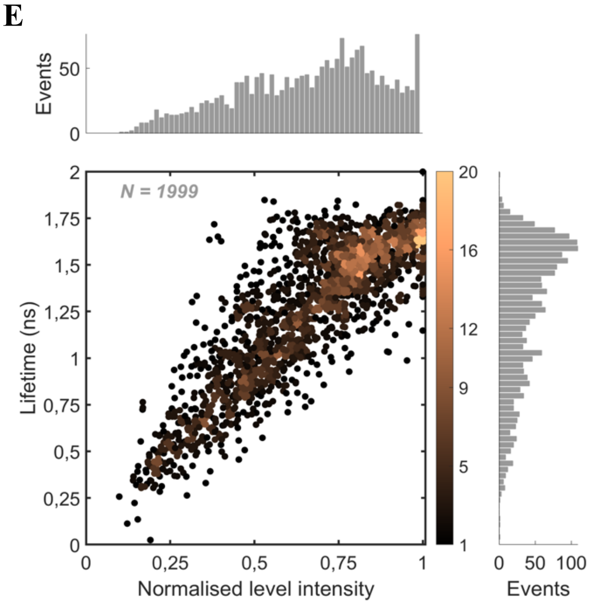

Fig. S3. Correlation between the normalised fluorescence intensities and lifetimes for WT-PB complexes at **A)** 272, **B)** 496, **C)** 800, **D)** 2480, and **E)** 4128 mW cm^{-2} illumination. N denotes the number of states, and the colour bars quantify the number of states per dot area.

Fig. S4

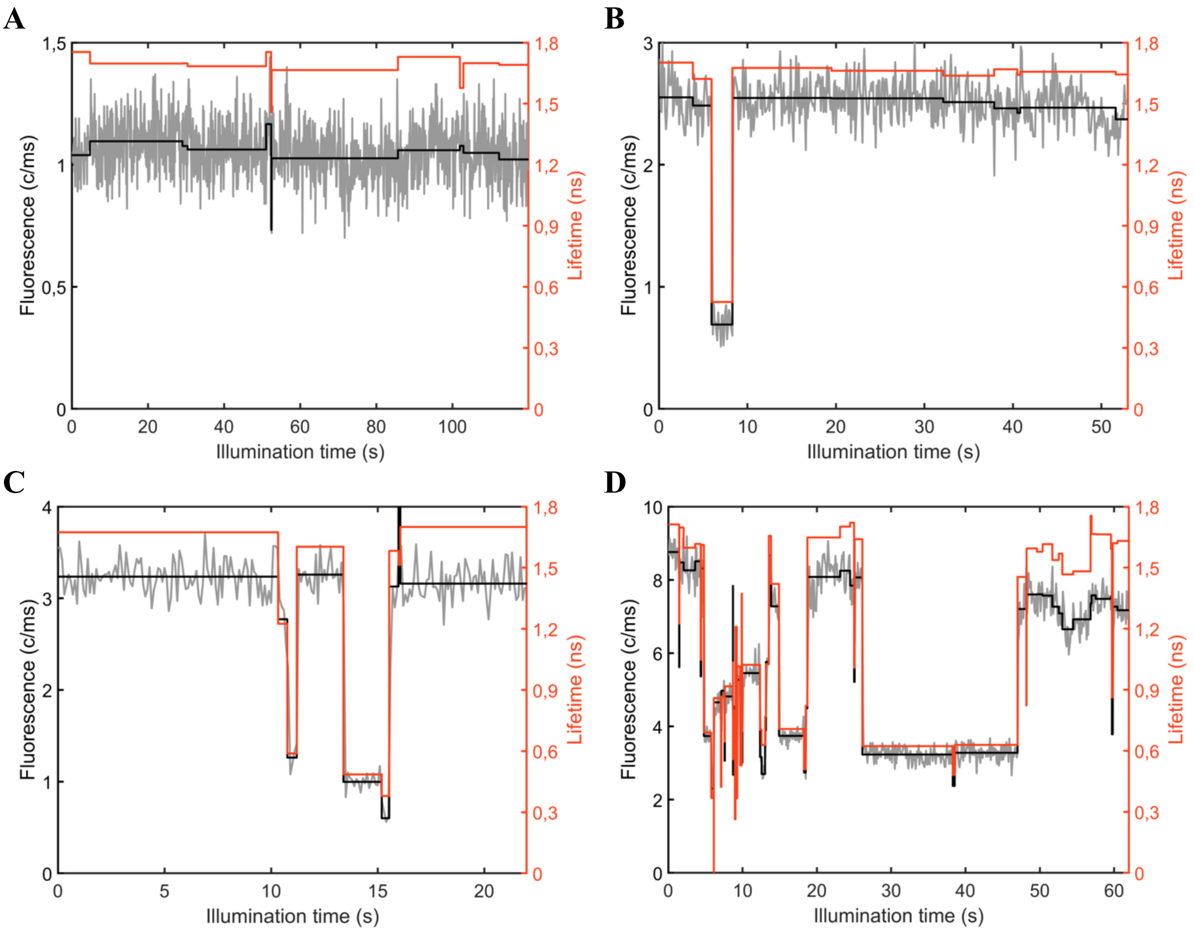

Fig. S4. Examples of emission and lifetime dynamics with increasing excitation rates for WT-PB at **A)** 496, **B)** 800, **C)** 1648, and **D)** 4128 mW cm^{-2} illumination. Fluorescence intensity traces (grey) with intensity levels (black) and fitted fluorescence lifetimes (red).

Fig. S5

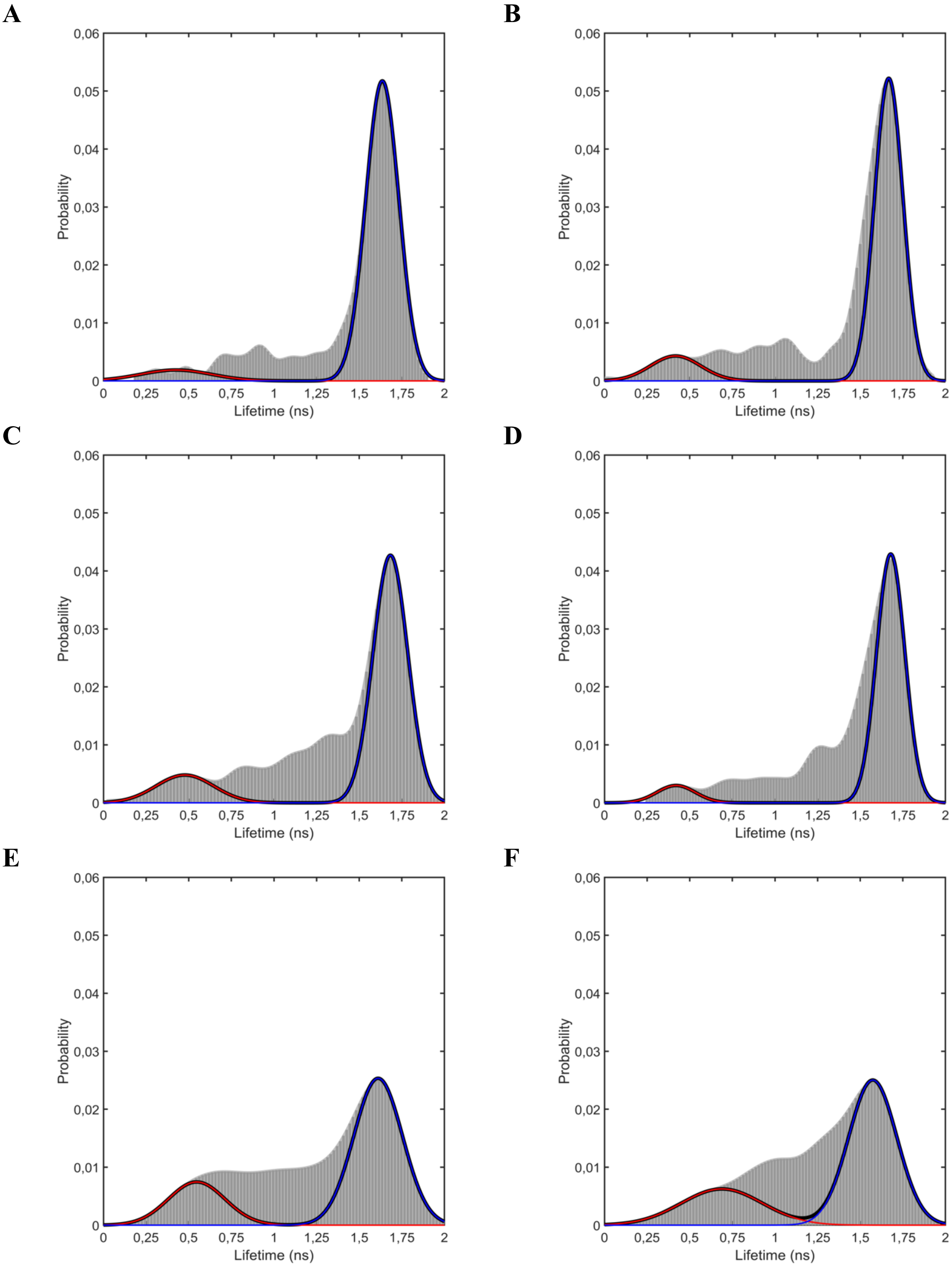

Fig. S5. Fluorescence lifetimes distribution for pre-screened WT-PBs at **A)** 272, **B)** 496, **C)** 800, **D)** 1648, **E)** 2480, and **F)** 4128 mW cm^{-2} illumination. To determine the threshold between the bright and dim states for two-state model analysis, each distribution was fitted with two Gaussians (red and blue). For both fits, half-Gaussians were initially fitted to the outer edges of the distributions and then mirroring second halves were added to complete both Gaussians. The saddle point between the Gaussians determined the threshold between bright and dim states.

Fig. S6

A

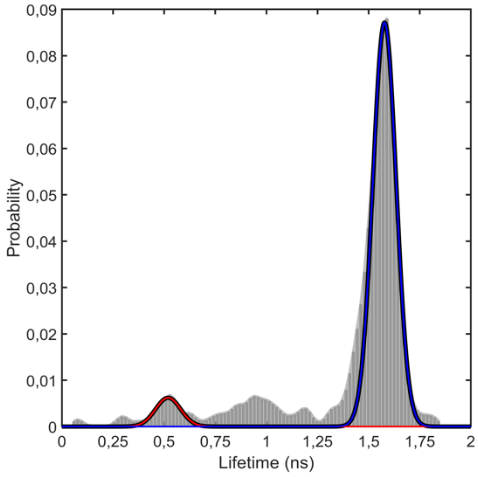

B

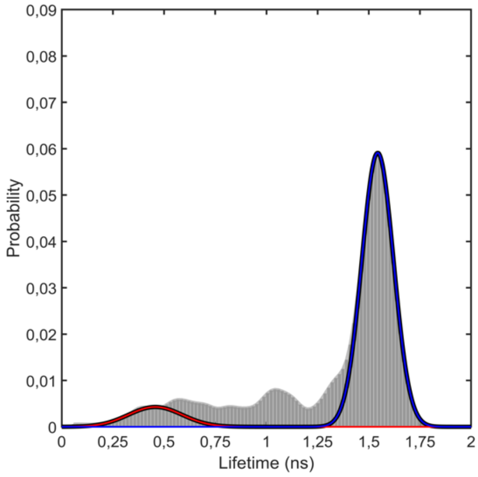

C

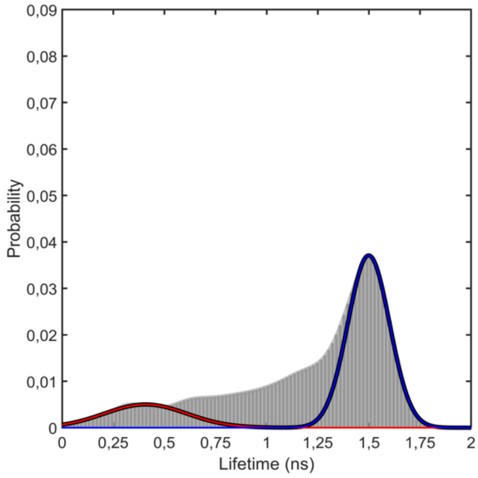

D

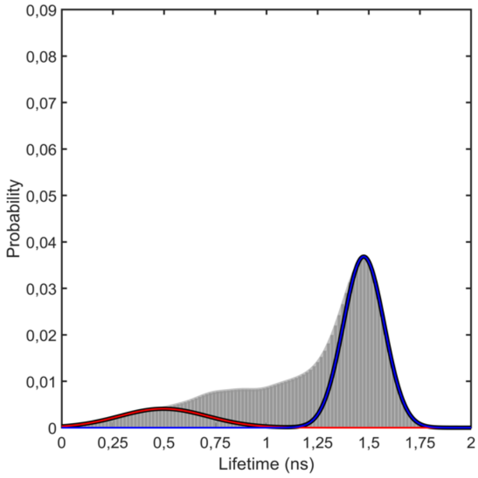

E

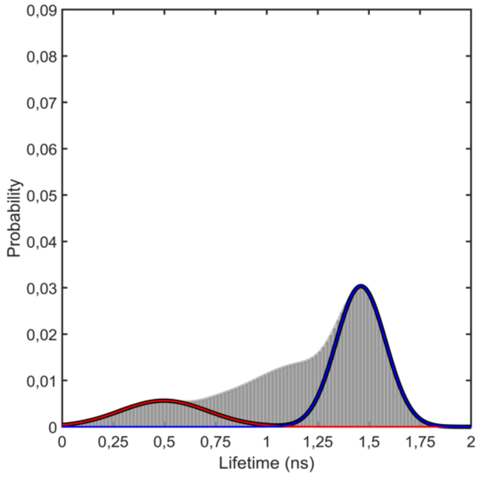

Fig. S6. Fluorescence lifetimes distribution for pre-screened ApcE-C190S-PB at **A)** 400, **B)** 896, **C)** 1648, **D)** 2448, and **E)** 4096 mW cm^{-2} illumination. To determine the threshold between the bright and dim states for two-state model analysis, each distribution was fitted with two Gaussians (red and blue) at the edges of the distributions (fitting like in Fig S5). The saddle point between the Gaussians determined the threshold between bright and dim states.

Fig. S7

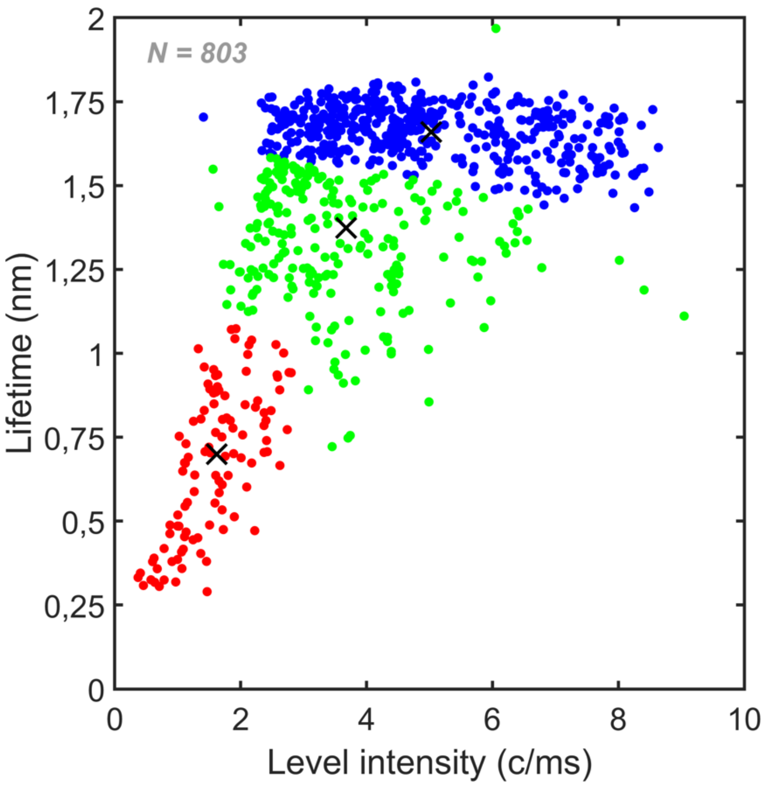

Fig. S7. Correlation between the non-normalised fluorescence intensities and lifetimes for 87 WT-PB complexes at 1648 mW cm^{-2} illumination (like in Fig. S1B). Data points were clustered according to a Gaussian mixture model, showing bright (blue), intermediate (green), and quenched (red) states.

Fig. S8

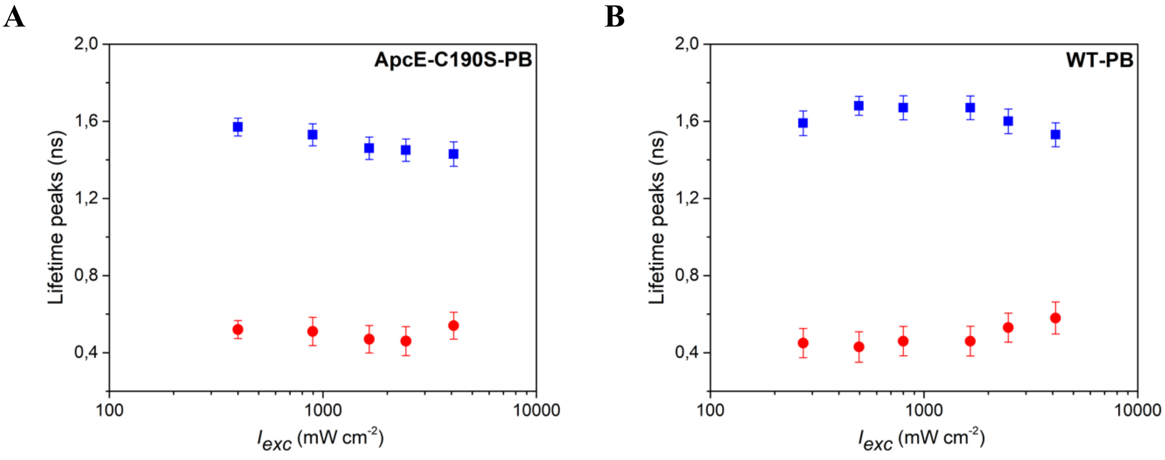

Fig. S8. Peak positions for Gaussians fitted to the distributions of lifetimes for **A)** ApcE-C190S-PB, and **B)** WT-PBs as shown in Figs S6 and S5, respectively.

Fig. S9

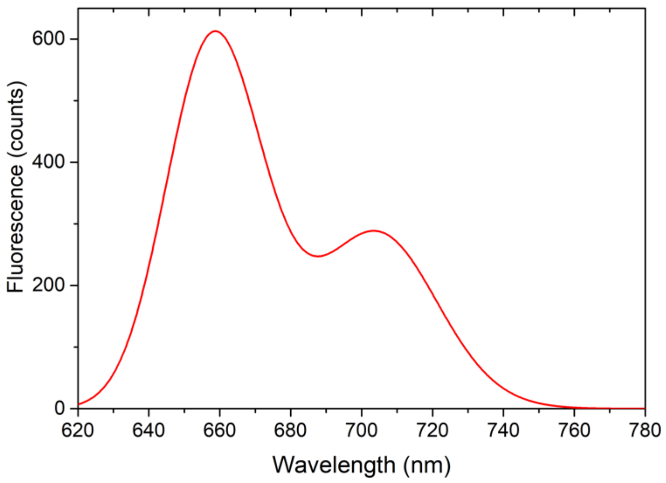

Fig. S9. An example of a spectrum from ApcE-C190S-PB displaying far-red emission. Similar spectra were previously recorded for WT-PB and signify the involvement of charge-transfer states in the light-induced energy quenching mechanism (for more information, see the main text and Krüger *et al.* 2019 & Wahadoszamen *et al.* 2020).

Fig. S10

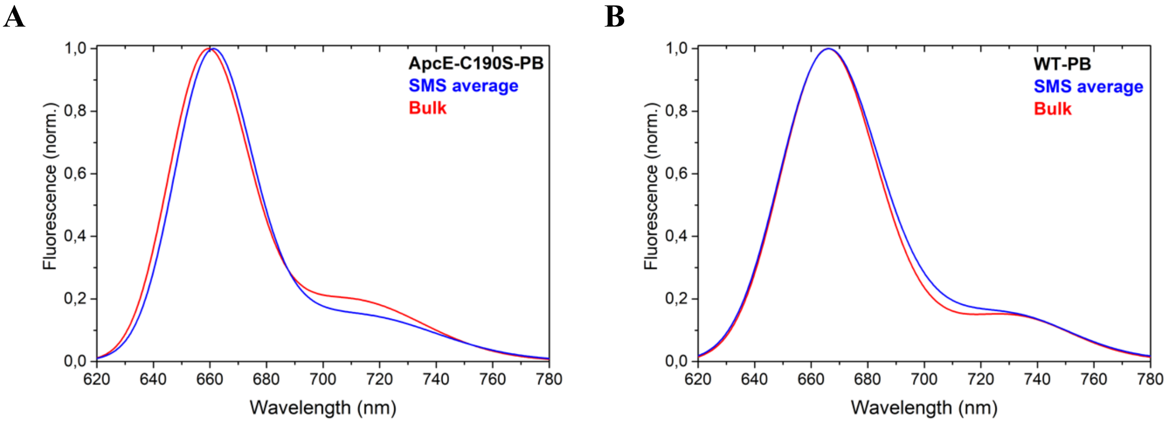

Fig. S10. Comparison between the average of all SMS spectra (of pre-screened complexes; blue) and bulk spectra (red) measured on the same spectroscopic setup for **A)** ApcE-C190S-PB and **B)** WT-PBs.

Fig. S11

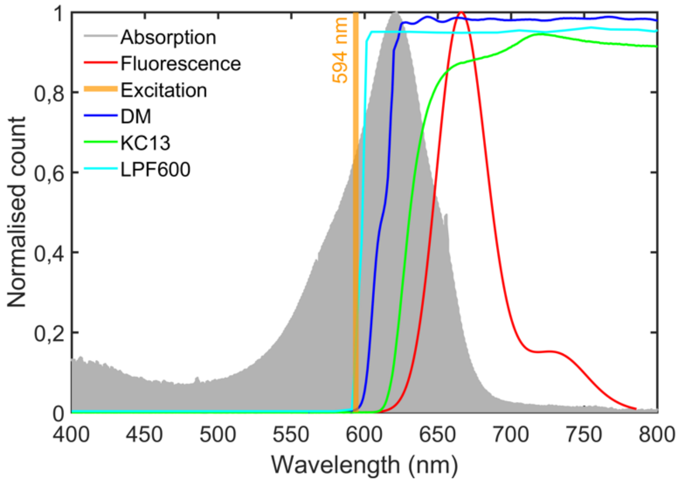

Fig. S11. PB absorption (grey) and emission spectra (red) compared with the excitation wavelength (vertical orange line at 594 nm) and transmission spectra of optical filters (blue, green, and cyan, with DM denoting the dichroic beam splitter). See the Methods section in the manuscript for details.

Fig. S12

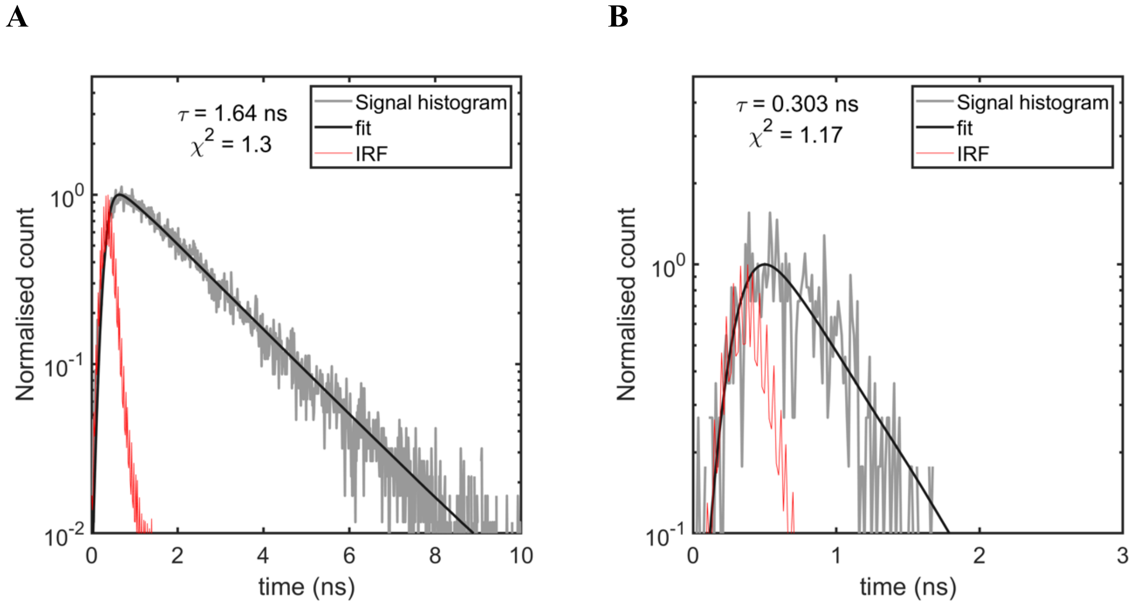

Fig. S12. Examples of fluorescence lifetime histograms (grey) deconvoluted with the measured IRF (red) and fitted with a monoexponential fluorescence decay function (black) for WT-PBs in **A)** unquenched, and **B)** quenched states.

Table S1

Table S1. Total number of complexes measured and selected for analysis at each experimental excitation intensity (see details in the main text).

	Excitation (mW cm^{-2})	Total no. of measured complexes	No. of manually selected complexes	No. of complexes after selection algorithm
WT-PB	272	145	122	85
	496	140	126	90
	800	130	113	92
	1648	139	121	87
	2480	172	157	112
	4128	146	132	84
ApcE-C190S-PB	400	224	181	144
	896	198	189	122
	1648	206	203	129
	2448	237	221	128
	4096	164	157	83

Table S2

Table S2. Saddle points for different illumination as obtained from Figs S5 and S6.

	Excitations (mW cm^{-2})	Saddle point (ns)	Average saddle point (ns) ± SD
WT-PB	496	1.35	**1.29 ± 0.07**
	800	1.31	
	1648	1.36	
	2480	1.19	
	4128	1.23	
ApcE-C190S-PB	400	1.37	**1.23 ± 0.10**
	896	1.28	
	1648	1.15	
	2448	1.23	
	4096	1.12	

Table S3

Table S3. Peak positions and the full width at half maxima (FWHM) for spectra shown in Figs 7 and S10.

	Spectrum	Peak position (nm)	FWHM (nm)
WT-PB	Average SMS – unquenched pre-screened complexes (Fig. 7B)	667.3	38.8
	Average SMS – quenched pre-screened complexes (Fig. 7B)	658.7	38.4
	Average SMS – all pre-screened complexes (Fig. S9B)	665.7	39.7
	Bulk (Fig. 7B & Fig. S9B)	665.8	39.1
ApcE-C190S-PB	Average SMS – unquenched pre-screened complexes (Fig. 7A)	662.9	30.9
	Average SMS – quenched pre-screened complexes (Fig. 7A)	654.8	29.4
	Average SMS – all pre-screened complexes (Fig. S9A)	660.8	31.6
	Bulk (Fig. 7A & Fig. S9A)	658.9	31.8
